# High-grade ovarian cancer associated H/ACA snoRNAs promote cancer cell proliferation and survival

**DOI:** 10.1093/narcan/zcab050

**Published:** 2022-01-14

**Authors:** Laurence Faucher-Giguère, Audrey Roy, Gabrielle Deschamps-Francoeur, Sonia Couture, Ryan M Nottingham, Alan M Lambowitz, Michelle S Scott, Sherif Abou Elela

## Abstract

Small nucleolar RNAs (snoRNAs) are an omnipresent class of non-coding RNAs involved in the modification and processing of ribosomal RNA (rRNA). As snoRNAs are required for ribosome production, the increase of which is a hallmark of cancer development, their expression would be expected to increase in proliferating cancer cells. However, assessing the nature and extent of snoRNAs’ contribution to cancer biology has been largely limited by difficulties in detecting highly structured RNA. In this study, we used a dedicated midsize non-coding RNA (mncRNA) sensitive sequencing technique to accurately survey the snoRNA abundance in independently verified high-grade serous ovarian carcinoma (HGSC) and serous borderline tumour (SBT) tissues. The results identified SNORA81, SNORA19 and SNORA56 as an H/ACA snoRNA signature capable of discriminating between independent sets of HGSC, SBT and normal tissues. The expression of the signature SNORA81 correlates with the level of ribosomal RNA (rRNA) modification and its knockdown inhibits 28S rRNA pseudouridylation and accumulation leading to reduced cell proliferation and migration. Together our data indicate that specific subsets of H/ACA snoRNAs may promote tumour aggressiveness by inducing rRNA modification and synthesis.

## GRAPHICAL ABSTRACT

**Graphical Abstract:**
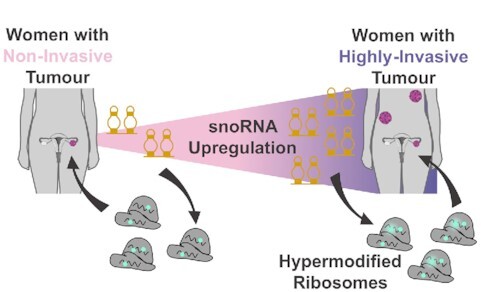
Identification H/ACA snoRNA signature of high-grade serous ovarian cancer.

## INTRODUCTION

Ovarian cancer is the most lethal cancer of the female reproductive system (1). Most ovarian cancers are epithelial in origin, 70% being high-grade serous carcinomas (HGSC) and 15% being serous borderline tumours (SBT) or tumours of low malignant potential (2,3). Epithelial ovarian cancer transforms normal ovaries from mesenchymal fibroblast endothelial cells surrounded by a layer of epithelial cells into a large mass of epithelial cells. While the origin of serous epithelial ovarian cancers is still being debated, it is believed that they originate from the fallopian tube epithelium (4). This hypothesis is supported by genome wide studies showing that SBT and HGSC are more like normal fallopian tube epithelium (NFTE) than normal ovarian tissues (5). This similarity makes NFTE the most suitable tissue for comparison with epithelial ovarian cancer.

HGSC is aggressive and mostly diagnosed at a late stage, while SBT is normally much less aggressive and often diagnosed when still restricted to the ovaries. Currently, the diagnosis of SBT and its differentiation from HGSC relies on histological criteria including epithelial cell proliferation, stratified epithelium, microscopic papillary projections, cellular pleomorphism, nuclear atypia, mitotic activities and the absence of stromal invasion, which differentiates it from invasive carcinomas (3,6–8). Relying on histological criteria makes accurate diagnosis difficult and prevents definitive estimation of the tumour's invasive potential and aggressiveness.

Currently there are two tests being used for ovarian cancer screens, transvaginal ultrasound (TVUS) and measurement of blood levels of CA-125 protein (9–11). TVUS only detects tumours of a certain mass and as such is not effective for very early stages of cancer development nor for differentiating between HGSC and SBT. On the other hand, CA-125 levels were found not to be useful for *de novo* screens since they are associated with a high rate of false positive results (12). Several markers for ovarian cancer are being developed or considered but most of these markers are yet to be validated as accurate early predictors of tumour invasive potential. One common problem with these putative markers, which mostly take the form of changes in the levels of protein coding mRNAs, is their reduced extra-cellular stability preventing detection in patients’ fluids (1,13). Therefore, there is a need for identifying different types of markers that are more stable than mRNA and have the capability of predicting tumour potential.

Mid-size non-coding RNAs (mncRNAs) are the most abundant and stable class of non-ribosomal RNA in human cells, and are much easier to quantify using RT-qPCR than small non-coding RNAs like microRNAs (miRNAs) (14). mncRNA vary in size between 40 and 400 nucleotides. Furthermore, many of these mncRNAs are among the most stable RNAs in serum and patients’ fluids, like urine samples (15,16). This makes mncRNA a prime target for the identification of non-invasive biomarkers for tumour aggressiveness potential. Indeed, many types of cancer have been associated with small nucleolar RNAs (snoRNAs), which is the second largest component of mncRNA after tRNA (14,17). snoRNAs are highly conserved and mostly function as a guide for the modification of ribosomal RNA (rRNA) and small nuclear RNA (snRNA). C/D box snoRNAs guide RNA 2′ O-ribose methylation, while H/ACA box snoRNAs guide pseudouridylation (18). These two classes of snoRNAs differ in their structure with the C/D box snoRNAs forming a loose stem loop, and the H/ACA box snoRNAs forming a tight, two-hairpin structure. The highly structured nature of these two snoRNA classes increases their stability but at the same time makes their detection using standard sequencing methods difficult and unreliable (17,19). Recently, we have developed a mncRNA sensitive pipeline that accurately quantifies the different species of mncRNAs including snoRNAs (17). Thanks to the use of a highly processive thermostable bacterial reverse transcriptase (RT) and a quantification pipeline that recognizes multimapped and intron embedded sequencing reads, we are now able to directly quantify snoRNA abundance in cancer tissues using RNA-seq (17,20).

In this study, we have taken advantage of our newly developed mncRNA sensitive sequencing pipeline to identify stable RNA species that could differentiate between HGSC and SBT tissues and serve as potential markers for tumour aggressiveness. RNAs showing differential expression in SBT and HGSC were selected and validated using RT-qPCR and used to create a molecular HGSC signature. The accuracy and precision of the new signature were validated using a mixed set of normal and tumour tissues and its relevance to the biology of ovarian cancer was examined in three different model ovarian cancer cell lines. Together our results identify three H/ACA snoRNAs as an effective HGSC signature and indicate that a subset of H/ACA snoRNAs may promote tumour aggressiveness by preventing apoptosis and inducing cell proliferation and invasion potential.

## MATERIALS AND METHODS

### Tissue sourcing RNA extraction, quality control and sequencing

The HGSC and SBT tissues were provided by the Ontario Tumour Bank, which is funded by the Ontario Institute for Cancer research, the FRQS tissue bank (Université de Sherbrooke), the Alberta Research Tumor Bank (ARTB) and the British Columbia Cancer Tumour Tissue Repository. The provenance and characteristics of each tissue sample used in this study are listed in Supplementary Table S1. Patients’ information, consents and certification were handled by the tumour banks and approved for the study described here after review and approval by the different tumour banks according to their material transfer agreement. Tissue-handling, protocols and procedures were reviewed and approved by the Comité d’éthique de la recherche du CIUSSS de l’Estrie- CHUS (CÉR) # 10-062, 2010-241. 30 mg of each tissue was homogenized in 1 mL of TRIzol Reagent (Ambion -ThermoFisher Scientific, USA) using a Polytron tissue homogenizer and the RNA was extracted as previously described (21). RNA integrity was assessed using the 2100 Bioanalyzer (Agilent Technologies, USA) and samples with RNA integrity index (RIN) >3 were used for subsequent analyses (Supplementary Table S1). To further confirm the RNA integrity, we compared the expression levels of a panel of housekeeping genes (MRPL19, RPPH1, RNU6 and PUM1) to *Escherichia coli* 23S rRNA spike-in (Roche Holding AG, Switzerland) using RT-qPCR (20 picograms of E. coli ribosomal RNA was added to each sample during the RT preparation) and tissues exhibiting housekeeping levels within 2 standard deviations from the average relative expression were kept. The HGSC and SBT tissue identity was evaluated by comparing the expression level of VEGF-A and WFDC2 using RT-qPCR and HGSC tissues showing increased expression of VEGF-A or WFDC2 relative to SBT were kept for further study. A cohort of randomly selected three HGSC and three SBT tissues were used for sequencing and the rest of tissues used for validation by RT-qPCR. TGIRT-seq libraries were constructed as previously described (17, for the unfragmented ribodepleted RNA TGIRT-seq, URT). Briefly, the prepared libraries were ribodepleted using the Illumina Ribo-Zero Gold rRNA Removal Kit (Human/Mouse/Rat). The ribodepleted RNA was purified with Zymo RNA Clean & Concentrator using a modified form of the protocol to conserve small RNAs (8× sample volume of 95–100% ethanol instead of 3×). Non fragmented, ribodepleted RNA was then used as input for TGIRT template-switching reactions with 1 μM TGIRT-III RT (InGex, LLC) for 15 min at 60°C, as previously described (17). After cDNA cleanup with QIAGEN MinElute Enzyme Reaction Cleanup kit, purified cDNAs are then ligated at their 3′ ends to a 5′-adenylated DNA oligonucleotide encoding the reverse complement of an Illumina Read 1 sequencing primer binding site. Finally, index and capture sequences required for Illumina sequencing are added by PCR (12 cycles). Libraries were size-selected with AMPure XP beads (Beckman Coulter) and library quality was evaluated using an Agilent 2100 Bioanalyzer. TGIRT-seq libraries were sequenced on an Illumina HiSeq 4000 or 2500 instrument (PE 2×150 and PE 2×125, respectively) at the Genome Sequencing and Analysis Facility (GSAF) at the University of Texas at Austin. ERCC spike-ins (17,20) were added and used as control for detection uniformity.

### RNA sequencing analysis

All datasets were analyzed using the same computational pipeline. Fastq files were checked for quality using FastQC v0.11.15 (22,23) and trimmed using Cutadapt v1.18 (24, http://journal.embnet.org/index.php/embnetjournal/article/view/200/479) and Trimmomatic v0.36 (25) (with TRAILING:30) to remove Illumina sequencing adapters and portions of reads of low quality, respectively. Reads were aligned using STAR v 2.6.1a (26) with the following options: –outFilterScoreMinOverLread 0.3, –outFilterMatchNminOverLread 0.3, –outFilterMultimapNmax 100, –winAnchorMultimapNmax 100, –alignEndsProtrude 5 ConcordantPair. Only primary alignments were kept using samtools view -F 256 (v1.5) (27). The libraries of samples LGr RT157 and LGr RT353 were split in multiple fastq files that were aligned separately then combined using samtools merge before quantification. The reference genome used was hg38 and the reference annotation to build the STAR alignment index was taken from Ensembl (v87) (28) supplemented with missing tRNAs and snoRNAs as described in (17). The gene quantification was done using CoCo correct count module (v.0.2.0) (29) with the option -s 1 and -p. DESeq2 (30) was used for differential expression analysis using raw counts obtained by CoCo. Log_2_fold change and Benjamini-Hochberg adjusted *P*-values were measured for each expressed gene. Equivalence between counts per million (CPM) and transcripts per million (TPM) was assumed since most RNAs detected using non-fragmented RNA libraries are usually shorter than 500 nucleotides. Volcano plots were generated using R package Enhanced Volcano (EnhancedVolcano, RRID:SCR_018931). Heatmaps were generated using R package gplots. ROC curves were generated using the CombiROC online software (31). 3D structure of the ribosome with the different rRNA modifications was generated using the online database of the ribosomal modifications (RRID:SCR_003097, (32)).

### Cell culture and transfection

The ovarian adenocarcinoma SKOV3ip1 cell line (RRID:CVCL_0C84) was grown in DMEM/F12 (50/50) medium supplemented with 10% (v/v) fetal bovine serum (FBS) and 2 mM l-glutamine. TOV112D endometrioid carcinoma cells (ATCC Cat#CRL-11731, RRID:CVCL_3612) were grown in OSE medium supplemented with 10% (v/v) FBS and 2 mM l-glutamine. OVCAR-3 ovarian cancer cells (CLS Cat#300307/p690_NIH:Ovcar-3, RRID:CVCL_0465) were grown in RPMI 1640 supplemented with 20% FBS (v/v) FBS and 2 mM l-glutamine. Cell passaging was performed as recommended by the American Type Culture Collection (ATCC), no more than 20 passages were carried out for each cell line. Each cell line was tested for mycoplasma contamination periodically (by qPCR method performed by the RNomics platform at the Université de Sherbrooke). Transfections were performed using Lipofectamine 2000 (Invitrogen) according to the manufacturer's protocol with 15 nM of either siRNA or antisense oligonucleotide (ASO) (containing five 2′*O*-methyl RNA bases, ten DNA bases with phosphorothioate bonds followed with five 2′*O*-methyl RNA bases). The sequence of the different ASO and siRNA and equivalent scrambled controls are listed in Supplementary Table S2. Cells were seeded at 5000 cells/well in 96-well plates and 300 000 cells/well in six-well plates. Plates were incubated for at least 48 h after transfection at 37°C under 5% CO_2_.

### Cell line RNA extraction and quantification using RT-qPCR

RNA was extracted using the RNeasy mini kit (Qiagen, Germany), following the manufacturer's guidelines (Qiagen, Germany) with exception of the RNA precipitation step. RNA was precipitated using 1.5 volume of 100% ethanol to conserve small size RNAs. RNA quantification, integrity and subunit ratio were assessed using the 2100 Bioanalyzer (Agilent Technologies, USA). cDNAs were produced using between 500 ng and 1 μg of RNA, MMULV- RT (Moloney Murine Leukemia Virus reverse transcriptase) (1 unit), RNaseOUT (20 units), dNTP (1 mM) and random hexamers (3 μM). The cDNA was diluted (3.33 ng/μl) and 10 ng was used for the qPCR reactions. qPCR reactions were performed in 96-well plates using the Eppendorf Realplex Mastercycler ep gradient and in 384-well plates using the Bio-Rad CFX RealTime System. The reactions were performed in 10 μl with 5 μl Bio-Rad iTaq Universal SYBR Green Supermix, 3 μl of diluted cDNA and specific primer pairs (200 nM) (Supplementary Table S3). No template and no RT reactions were used as negative controls. The normalized relative expression was calculated using the ΔΔCq where Cq is the quantification cycle. The resulting abundance distributions were compared using a two-tailed Student's *t*-test assuming unequal variance.

### Western blot

Cells were collected, pelleted and lysed in 50 μl lysis buffer (150 mM NaCl, 50 mM Tris–HCl (pH 8), 0.5% NP40, 0.5% sodium deoxycholate, 0.1% sodium dodecyl sulfate, 10 mM sodium pyrophosphate and 50 mM EDTA) containing protease inhibitor cocktail (Complete, EDTA-free, Roche Diagnostics Indianapolis, IN) for 1 h at 4°C in a tube rotator and centrifuged for 20 min. Protein concentration was determined using Bradford Protein Assay. Equivalent 10 μg protein samples were solubilized in Laemmli buffer, (62.5 mM Tris–HCl (pH 6.8), 10% glycerol, 5% β-mercaptoethanol and 2.3% SDS) denatured 3 min at 95°C and loaded onto 8% (EIF3A) and 12% (EIF4A2) gels. Electrotransfer to Hybond-ECL (GE Healthcare, Piscataway, NJ) was performed for 2 h at 100 V. Membranes were blocked for 1 h at room temperature in TBS containing 0.1% Tween-20 and 5% skimmed milk. Membranes were hybridized overnight at 4°C in blocking solutions with agitation on a Nutator (Clay Adams Brand), with primary antibodies diluted at 1:1000 (Anti-EIF4A2: Abcam Cat#ab31218, RRID:AB_732123 and Anti-EIF3A: Abcam Cat#ab86146, RRID:AB_2096634) and 1:2000 (anti-GAPDH: Novus Cat#NB300-221, RRID:AB_10077627). Membranes were washed in TBS containing 0.1% Tween-20. Secondary antibodies were diluted in blocking solution at 1:1000 (anti-rabbit IgG: GE Healthcare Cat#NA934, RRID:AB_772206) and 1:2000 (Anti-mouse IgG: GE Healthcare Cat#NA931, RRID:AB_772210) and membranes were hybridized 90 min at room temperature with agitation on a Nutator (Clay Adams Brand). Membranes were washed and proteins were detected by chemiluminescence with Clarity Western ECL (Bio-Rad, Saint-Laurent, QC), using LAS 4000 (GE Healthcare, Mississauga, ON).

### Real time cell growth assay

XCELLigence (ACEA Biosciences Inc., San Diego, CA, USA) was used to analyze cell adherence in real time (33). E-plates were used to assess cell viability, while CIM plates were used to assess cell migration and invasion. For cell viability, 50 μl of complete culture medium was added to each well of an E-plate. The plate was incubated for 30 min at 37°C with 5% CO_2_. Then, a concentration of 5000 cells per 50 μl was added to each well. Measurements were taken every 10 min for 3 h before the transfection. Following transfection, measurements were taken every 10 min up to at least 70 h for a maximum of 90 h. For cell migration assay, CIM plates were pre-assembled by adding 160 μl of medium containing serum to each well of the bottom chamber of the CIM plate, the upper chamber was assembled on top and 50 μl of serum free medium was added to each well. The plate was incubated for one-hour at 37°C with 5% CO_2_. The same procedure was used for cell invasion assay, except Matrigel gel (Corning, 356234) was added to coat the bottom of the upper chamber at a concentration of 1:40. Essentially as described before 50 μl of Matrigel was diluted and added to the upper chamber and 30 μl was removed to ensure proper coating of the chambers (33). The upper chamber was incubated for 5 h to ensure polymerization of the Matrigel. Bottom and upper chambers were assembled as mentioned previously. For both cell migration and invasion assays, cells were collected and 100 μl were seeded at a concentration of 50 000 cells/well (migration assay) or 25 000 cells/well (invasion assay) 24 h following transfection in six-well plates. Measurements were taken every 10 min up to at least 70 h for a maximum of 90 h. Each experiment included three technical and three biological replicates.

### Multiplex apoptosis and necrosis assay

The multiplex phenotypic assay was carried out as previously described (34). The medium was removed 48h after transfection in 96-well plates and 50 μl coloration mix was added to each well. The coloration mix was composed of the four following dyes at specific concentrations: Hoechst (32 μl of stock solution at 100 μg/μl, Invitrogen), Calcein AM (with a 0.5 μM final concentration, Invitrogen), Alexa Fluor 647 (11.4 μl of stock solution, Invitrogen), Propidium Iodide (4 μl of stock solution at 1 mg/ml, Invitrogen) in 4 ml of Annexin V binding buffer (10 mM HEPES, 140 mM NaCl, 2 mM CaCl_2_, pH 7.4). The plate was incubated for 30 min at 37°C and Annexin V binding buffer containing the dye was removed and 50 µL of new Annexin V binding buffer was added. The plate was then imaged using the Perkin Elmer Operetta. Nine pictures of different regions of each well were made and the images imported into the Columbus software (PerkinElmer, Waltham, MA, USA). Each experiment included four technical and three biological replicates.

### Cell proliferation assay

BrdU (5-bromo-2-deoxyuridine) (Millipore, 203806) was used to assess cell proliferation and the assay was performed in 96-well plates, 48 h after transfection. BrdU (0.75 mM) was added to cells and incubated for 90 min at 37°C, followed by washing with PBS. To fix and permeabilize the cells, four different washing steps were conducted starting with paraformaldehyde (7.4%), then PBS, then Triton X-100 diluted in PBS (0.1%) and finishing with PBS. Bovine serum albumin 2% (BSA) in PBS was added followed by an incubation of 45 min at room temperature. DNase I (300 μg/ml) was added, followed by an hour incubation at 37°C. The primary antibody, anti-BrdU (1:500) (Millipore Cat#MAB3222, RRID:AB_94758), was added and the plate incubated at room temperature for 1 h. The cells were then washed twice with PBS, and the secondary antibody was added, goat anti-mouse IgG (H + L) Alexa fluor 488 (1:250 in 2% BSA) (Thermo Fisher Scientific Cat#A-11029, RRID:AB_2534088), as well as Hoechst stain (800 ng/ml) (Thermo Fisher Scientific, Waltham, MA, USA). Cells were then incubated at room temperature in the dark for 45 min. A final wash of 100 μl PBS was completed, and the plate was read with the Perkin Elmer Operetta. Nine pictures of different regions of each well were taken and the images imported into the Columbus software (PerkinElmer, Waltham, MA, USA). Each experiment included four technical and three biological replicates.

### Flow cytometry assay

Flow cytometry assay was performed in six-well plates. Forty-eight hours after transfection, medium was removed and collected. Cells were PBS-washed, and trypsinized. Cells were collected and pelleted for 5 min at 100g, then suspended in ice-cold PBS. Cells were fixed by addition of cold ethanol (100% at −20°C), and then incubated at −20°C for a minimum of 30 min up to two weeks. Cells were again pelleted for 5 min at 400g, followed by 30 s at 16 200g and washed in PBS. The double centrifugation and the PBS washing was repeated three times. RNase A (100 μg/ml in PBS) was added, and the cells were incubated 5 min at room temperature. Propidium iodide was then added, and the solution again incubated for 30 min at room temperature. Cells were then counted by FACS (Flow Cytometer BD Fortessa). The experiment was carried out in three biological replicates.

### Wound healing assay

Wound healing assays were performed 48 h after transfection in 96-well plates. Culture medium was removed from the well and a scratch was performed in the middle of the well with a 200 μl tip. Medium was added containing hydroxyurea (60 mM) to inhibit cell proliferation. Using the Cloneselect apparatus (Molecular Devices) pictures of each well were taken 24 and 48 h after the scratch. The experiment was carried out in three biological replicates.

### CMC-RT and ligation assisted PCR analysis of pseudouridylation (Ψ) modification (CLAP) assay

CLAP assays were performed essentially as described earlier (35). Briefly, 25 μg of RNA was used for the CMC-treated (15 μg) and CMC-non treated (10 μg) samples in which TEU buffer (50 mM Tris–HCl, pH 8.3, 4 mM EDTA, 7 M urea) was added either containing (CMC-treated) or not (CMC-non treated) 1 M CMC (ThermoFisher, USA). The samples were incubated at 30°C for 16 h. Crush soak buffer (50 mM KOAc, 200 mM KCl, pH 7) was added with glycogen (5 μg/μl). RNA was recovered by adding ethanol (2.7×) and incubated at 80°C for 1 h. RNA samples were centrifuged, washed with ethanol 70% and incubated for 1 h at 80°C, those steps were repeated twice. RNA samples were centrifuged and resuspended in reverse buffer (50 mM Na_2_CO_3_, 2 mM EDTA, pH 10.4) and incubated at 37°C for 6 h. RNA samples were diluted in crush-soak buffer and ethanol precipitation steps were repeated. RNA samples were resuspended in sterile water. RNA samples were phosphorylated by adding T4 PNK reaction buffer, ATP, RNase inhibitor and T4 PNK (NEB, USA) and incubated at 37°C for 30 min. RNA samples were ligated with a blocker RNA oligo (/5AmMC6/rArCrCrCrA, IDT) by adding T4 RNA Ligase Reaction Buffer, ATP, RNase inhibitor, DMSO, sterile water and T4 RNA ligase I (NEB, USA) and the reactions were incubated at 16°C for 16h. EDTA was added to stop the reaction. 3 μl of the CMC ligation mixture were used to perform reverse transcription using the AMV reverse transcriptase where annealing buffer (250 mM Tris–HCl, 480 mM KCl, pH 7.4) was added and 0.5 μM of the target specific RT primer (Supplementary Table S3). The samples were incubated at 93°C for 2 min followed with 3 min at room temperature. The AMV RT reaction mixture was added containing the AMV RT (NEB, USA), AMV buffer, 1 mM of each dNTP. The samples were incubated at 42°C for 1 h followed with 5 min at 85°C and finished by putting on ice. RNase H (NEB, USA) was added to the samples and incubated at 37°C for 20 min. RNase H was inactivated by incubating the samples at 85°C for 5 min and finished by putting on ice. The specific 3′ adaptor/splint oligo (Supplementary Table S3) mixture was added to each sample and incubated at 75°C for 3 min and 3 min at room temperature. The ligation mixture (T4 DNA ligase (NEB, USA), T4 DNA ligase buffer and 50% DMSO) was added to each sample and incubated at 16°C for 16 h. The reaction was inactivated at 65°C for 10 min and finished by putting on ice. The samples were amplified by PCR using the Q5 high fidelity DNA polymerase. The specific forward and reverse primers were added with the cDNA and the Q5 DNA polymerase mixture (Q5 buffer, Q5 high GC enhancer, 200 μM of each dNTP and Q5 high-fidelity DNA polymerase (NEB, USA). The PCR was performed following the manufacturer's conditions for 25 cycles. The samples were then examined and amplified amplicons quantified using the Perkin Elmer LabChip GX Touch HT apparatus. To calculate the percent of modified nucleotides, the peak heights (modified and unmodified) were quantified, and the following formula was used: 100 × modified peak/(modified + unmodified peaks). A negative control (non-CMC treated RNA) was used where only the unmodified peak can be detected. Technical triplicates were averaged.

## RESULTS

### Identification of high-grade ovarian cancer associated mncRNA

**Figure 1.**
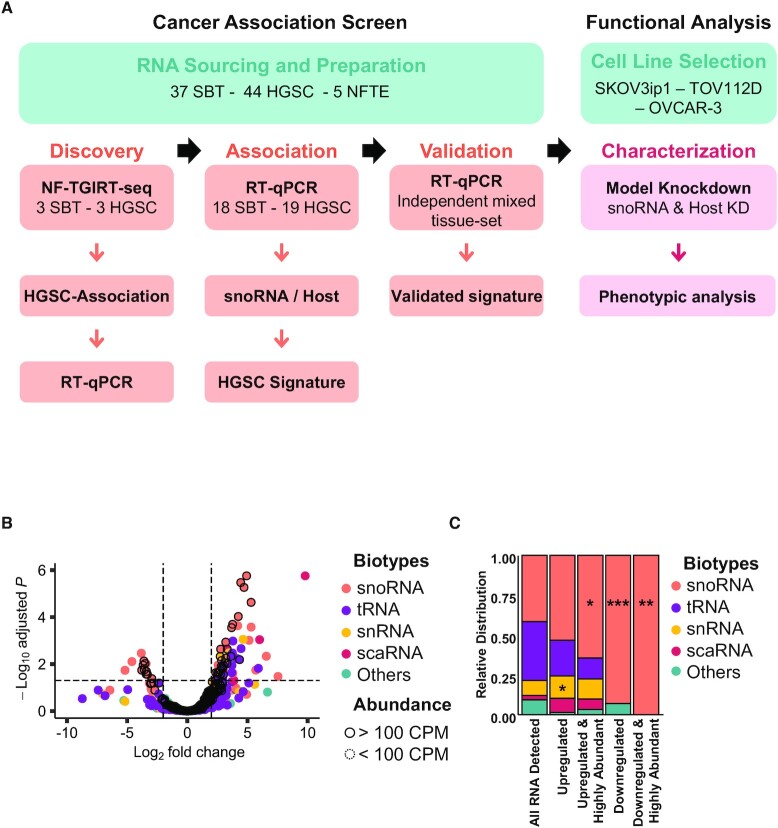
snoRNA expression is specifically modified in high-grade ovarian cancer. (**A**) Strategy for the identification of high-grade ovarian cancer associated mid-size non-coding RNA. Total RNA of three HGSC and three SBT was sequenced using non-fragmented TGIRT-seq (NF-TGIRT-seq). RNAs that are upregulated by >2-fold in HGSC as compared to SBT with a Benjamini-Hochberg adjusted *P*-value ≤0.05 were verified using RT-qPCR in the same sequenced tissues. snoRNA association with HGSC was evaluated using an independent set of 37 tissues and snoRNA signature identified. The signature capacity to discriminate between HGSC, SBT and normal fallopian tube epithelium (NFTE) tissues was evaluated using an independent set of mixed tissues. To functionally analyze the validated snoRNA signature, individual snoRNAs were knocked down using antisense oligonucleotides (ASOs) and evaluated for function using three model ovarian cancer cell lines. (**B**) snoRNAs are the most abundant class of HGSC-associated mncRNAs as compared to SBT. The log_2_ fold change in abundance of HGSC RNA relative to SBT RNA as measured by NF-TGIRT-seq was plotted against the –log_10_ adjusted *P*-value. The most significantly down- and up-regulated mncRNAs in HGSC are respectively in the upper most left and right quadrants of the volcano plot. Dots highlighted with a dark contour indicate RNAs expressed at more than 100 CPM. The biotype of the different RNA classes is indicated on the right. (**C**) snoRNAs are specifically dysregulated in HGSC as compared to SBT. The relative distribution of RNAs that are detected (>1 CPM), differentially expressed (– or + log_2_ fold change and adjusted *P*-value of at most 0.05), and highly abundant (>100 CPM) in HGSC is shown as stacked bar plots. The name of the different RNA classes is indicated on the right. The stars indicate the significance as measured by Chi-square tests with *P*-values of 0.03 (*), 0.02 (*), 0.0003 (***) and 0.001(**) from left to right when comparing the relative enriched proportion of each mncRNAs between the full dataset (all RNA detected) and the subsets of interest.

To identify potential biomarkers of tumour aggressiveness, we compared the expression pattern of mncRNAs in HGSC and SBT, which are similarly composed of mostly epithelial cells, thus minimizing the risk of cell type bias (36). The screening process was performed in three stages (Figure 1A). The first discovers mncRNA that are differentially expressed in HGSC and SBT, the second associates mncRNA with HGSC and the third validates the HGSC signature. In the discovery phase, we used our recently developed mncRNA sensitive sequencing method (non-fragmented TGIRT-seq) to identify mncRNAs that are differentially expressed between three SBT and three HGSC tissues. To confirm the association of the newly discovered differentially expressed RNA with HGSC, we examined their expression in an independent set of 18 SBT and 19 HGSC tissues using RT-qPCR. The potential snoRNA signature identified in the second stage was challenged using an independent set of mixed tissues containing 16 SBT, 22 HGSC and 5 normal fallopian tube epitheliums (NFTE). All extracted RNA resulted in a stable and consistent RT-qPCR amplification of four different housekeeping genes (MRPL19, RPPH1, RNU6 and PUM1) confirming the quality and integrity of the RNA. The identity of the selected tissues was confirmed both by the pathology report and the expression levels of VEGF-A (37–39) and WFDC2 (40,41), which were shown to be upregulated in HGSC as compared to SBT (Supplementary Figure S1).

As indicated in Figure 1B, all four main biotypes of mncRNA (snoRNA, tRNA, snRNA and scaRNA) were detected by sequencing and the majority were similarly expressed in both SBT and HGSC tissues. This suggests that the overall expression program of mncRNAs is not modified in HGSC when compared to SBT. Instead, a small subset of 58 mncRNAs present at >1 CPM was differentially expressed in HGSC and SBT with a log_2_ fold change of >2 and a Benjamini-Hochberg adjusted *P*-value of <0.05 (Figure 1B). The majority of the RNAs that were differently expressed in HGSC and SBT were tRNAs or snoRNAs. To ensure stable detection of any potential markers by RT-qPCR across clinical samples with varying RNA quality we selected RNA with >100 CPM for further study (Supplementary Figure S2A). In our hands, this arbitrary cut-off reduces variations in RNA detection. Surprisingly, most of the differentially expressed RNAs present at >100 CPM were snoRNAs (Figure 1C). Indeed, the results indicated that 65% of all highly expressed mncRNAs that are upregulated and 100% of those that are downregulated in HGSC relative to SBT were snoRNAs. Strikingly, snoRNAs were the only biotype showing statistically significant enrichment in abundance proportion between all mncRNAs detected and the dysregulated (either up- or downregulated) and highly abundant categories between HGSC and SBT (Figure 1C). These data indicated that the expression of snoRNAs is specifically modulated in HGSC when compared to SBT.

### H/ACA and C/D box snoRNAs are differentially regulated in high-grade ovarian cancer

**Figure 2.**
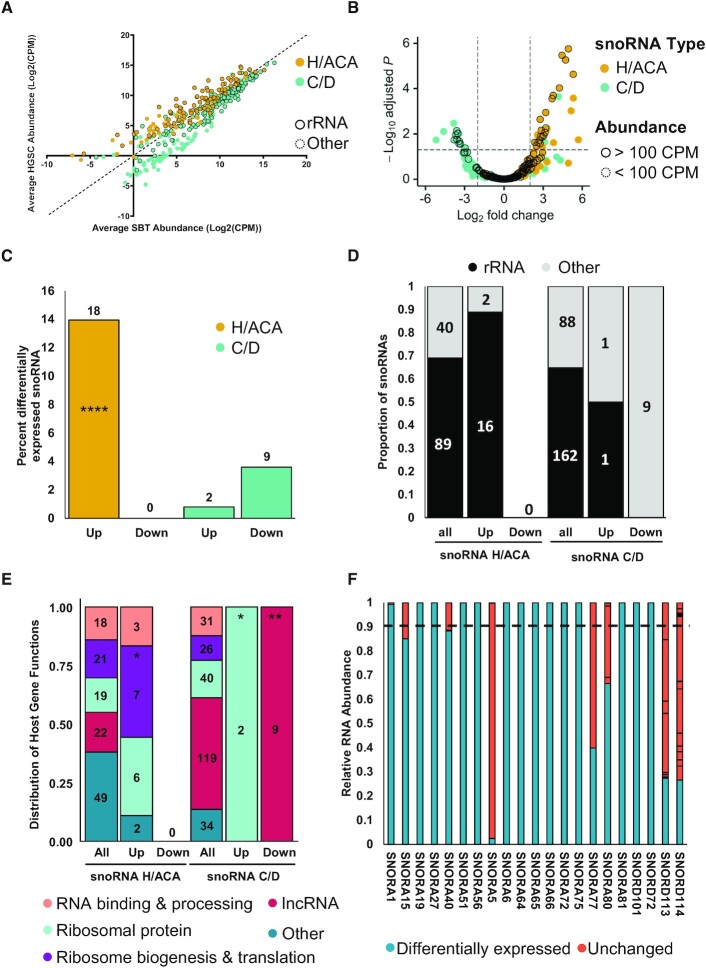
H/ACA and C/D box snoRNAs are differentially expressed in HGSC as compared to SBT. (**A**) H/ACA and C/D box snoRNAs display distinct abundance patterns in HGSC and SBT. The abundance of H/ACA and C/D snoRNAs as determined by NF-TGIRT-seq was compared using a scatter plot. C/D snoRNAs are shown in cyan and H/ACA snoRNAs are shown in yellow for panels (A) to (C). Dots highlighted with a dark contour indicate snoRNAs with predicted rRNA modification targets. (**B**) H/ACA snoRNAs are the most upregulated class of snoRNAs in HGSC as compared to SBT. The log_2_ fold change of snoRNA abundance in HGSC relative to that of SBT was plotted against the –log_10_ adjusted *P*-value. The most significantly down- and up-regulated mncRNAs in HGSC are in the upper most left and right quadrants of the volcano plot, respectively. Highlighted dots indicate RNAs expressed above 100 CPM. (**C**) C/D snoRNAs are mostly down regulated in HGSC as compared to SBT. Bar graph showing the percent of snoRNAs that are highly expressed (expressed above 100 CPM) and either up or down regulated in HGSC. The numbers above the bars indicate the number of snoRNAs that fall within that category. The proportion of upregulated H/ACA snoRNAs is significantly higher than the proportion of C/D snoRNAs in HGSC (Fisher's exact test ****P < 0.0001). (**D**) Most HGSC-associated snoRNAs target ribosomal RNA modifications. The proportion of snoRNAs that target rRNA modifications is indicated for both up and down regulated snoRNAs in the form of a stacked bar graph. The numbers inside the bars indicate the number of snoRNAs that fall within that category. All, up and down indicate respectively all detected snoRNAs (at least 1 CPM in one or more tissue), upregulated or downregulated in HGSC. (**E**) Most upregulated snoRNAs are expressed from host genes coding for proteins associated with ribosome biogenesis and translation. The function of the host genes harboring all detected, upregulated, and downregulated snoRNAs is indicated in the form of a stacked bar graph. The categories of the host gene functions are indicated on the bottom. The proportion of snoRNAs encoded in host genes with specific functions were compared between all detected snoRNAs and those in subgroups of interest, per snoRNA class. The star (*) indicates *P*-values ranging from 0.05 to 0.03 while ** indicate a *P*-value of 0.006. (**F**) Most HGSC-associated snoRNAs are expressed from a single expressed gene copy. The relative abundance of the RNA generated from the different gene copies coding for each HGSC-associated snoRNA is indicated in the form of a bar graph. The differentially expressed snoRNA copies are indicated in blue and those produced from other copies indicated in pink. The dashed line is the cut-off at 90%.

The observed changes in the abundance of snoRNAs between the two tissue types may stem from a general dysregulation of the ribosome biogenesis machinery or gene-specific modulation of gene expression. To differentiate between these two possibilities, we compared the abundance of H/ACA and C/D snoRNAs in HGSC and SBT tissues. Surprisingly, the results indicated that H/ACA and C/D snoRNAs were inversely regulated in HGSC tissues as compared to SBT (Figure 2A and B). The overall expression of H/ACA snoRNAs tended to increase in HGSC, while the expression of C/D snoRNAs tended to decrease (Figure 2A). Analysis of the amplitude and statistical significance of the change in the abundance of snoRNAs indicated that, while a subset of C/D snoRNAs were significantly downregulated most of the H/ACA snoRNAs were upregulated in HGSC as compared to SBT (Figure 2B, Supplementary Figure S2B). Indeed, no H/ACA snoRNA was significantly downregulated in HGSC and only two C/D snoRNAs were upregulated and highly expressed in HGSC as compared to SBT (Figure 2C). The nine downregulated C/D snoRNAs represented 3.6% of the expressed C/D snoRNAs and were all part of the highly duplicated SNORD113-SNORD114 families embedded within the introns of lncRNA MEG8, which was previously linked to pancreatic and lung cancer epithelial to mesenchymal transition (EMT) (42,43). The two C/D snoRNAs that were highly expressed and upregulated in HGSC (SNORD72 and SNORD101) represented only 0.8% of all expressed C/D snoRNAs and were embedded in two different ribosomal protein host genes (RPL37 and RPS12). This indicated that high-grade ovarian cancer associates with changes in the expression of a small subset of C/D snoRNAs that are mostly more highly expressed in borderline tumours.

Unlike C/D snoRNAs, none of the H/ACA snoRNAs were downregulated in HGSC and 14% of all expressed H/ACA snoRNAs (18 snoRNAs) were upregulated in HGSC as compared to SBT (Figure 2C). The higher proportion of upregulated H/ACA in HGSC compared to C/D was found to be statistically significant (Fisher's exact test, *P* < 0.001). Interestingly, the majority (16/18) of upregulated H/ACA snoRNAs were predicted to guide rRNA modifications, indicating that the upregulation of these snoRNAs may signify changes in ribosome biogenesis and/or function (Figure 2D). Strikingly, the opposite trend was found for the C/D snoRNAs with dysregulated expression in HGSC (either up or down regulated), as the majority (10/11) were not predicted to guide rRNA modifications, suggesting different roles for the two snoRNA classes in ovarian cancer tumorigenesis. Consistent with the findings for HGSC upregulated H/ACA snoRNAs, a significant proportion (13/18) are embedded within ribosome related genes, most specifically genes which encode ribosomal proteins, or proteins involved in ribosome biogenesis and translation (Figure 2E). Many snoRNAs are known to be produced from multicopied genes (44) and thus more than one gene can generate the exact same snoRNA or a snoRNA with very close sequence. Such snoRNAs with very close sequence are referred to as snoRNAs of the same family. Care must thus be taken when quantifying same-family snoRNAs using RNA-seq and qPCR. Interestingly, most snoRNAs showing modified expression in HGSC as compared to SBT were primarily generated from a single expressed gene, except for the highly duplicated SNORA5, SNORD113 and SNORD114 (Figure 2F). Of the 20 upregulated snoRNAs (2 C/D and 18 H/ACA snoRNAs), 14 snoRNAs represented at least 90% of the sum of the abundance of all copies of their family (2 C/D and 12 H/ACA snoRNAs). These 14 mostly monogenic snoRNAs are more likely to be homogeneously regulated in HGSC and thus were selected for further study.

### Identification of HGSC snoRNA signatures

**Figure 3.**
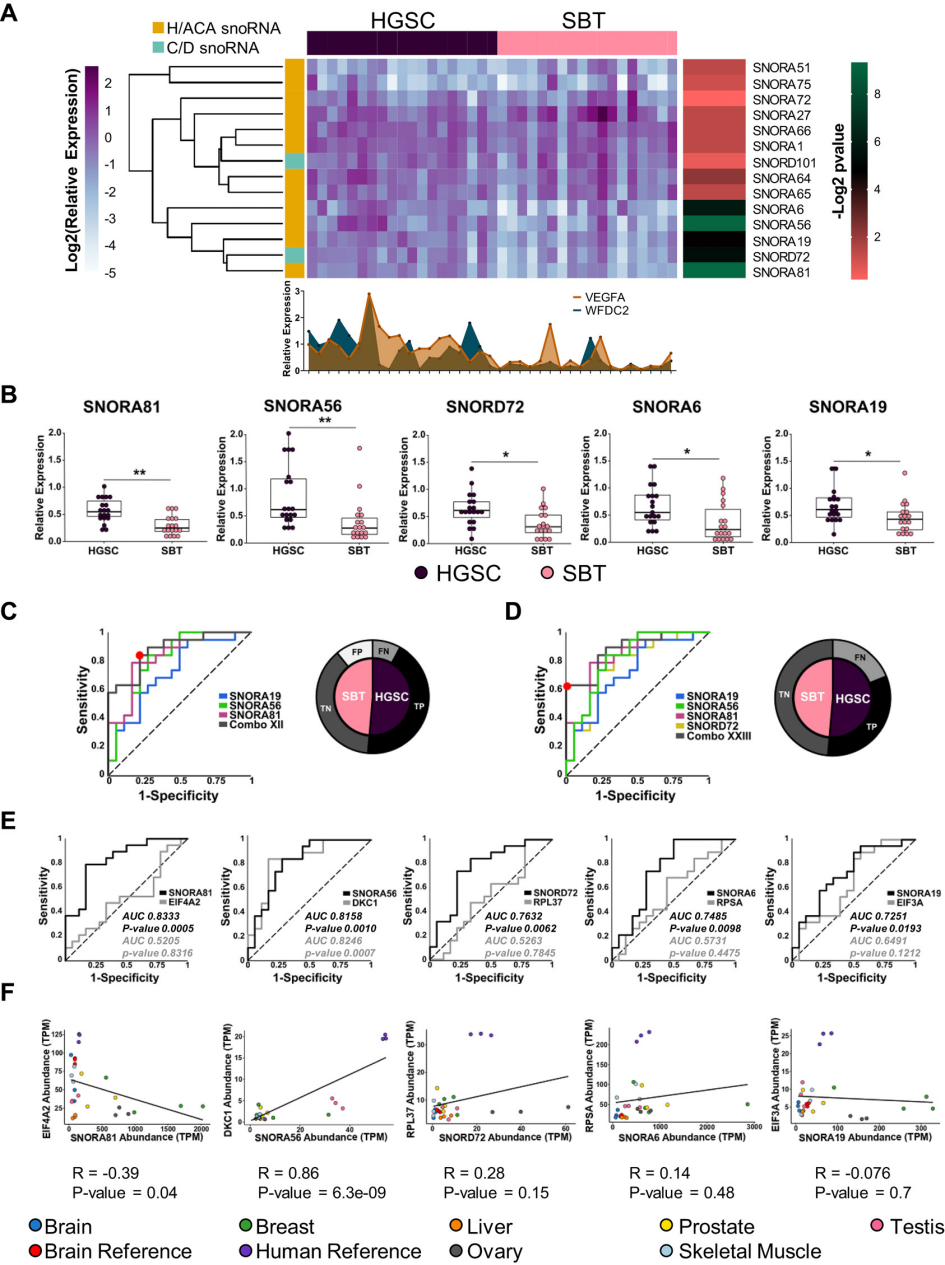
Identification of a HGSC snoRNA signature. (**A**) Five snoRNAs accurately discriminate between HGSC and SBT derived from independent patients’ cohorts. The relative abundances of the 14 HGSC-associated snoRNAs identified by sequencing were examined in a set of 37 tissues using RT-qPCR. Data were normalized relative to the mean amount of MRPL19 mRNA and a spike-in of *E. coli* 23S rRNA, and the log_2_ of the relative expression is presented in the form of a horizontally clustered heatmap. The snoRNA class is indicated on the left, the identity of the tissue of origin shown on the top and the significance (-log2 of Benjamini-Hochberg adjusted *P*-value) of differential expression between HGSC and SBT is shown on the right. For comparison, the histogram shown at the bottom indicates the relative expression of two known HGSC molecular markers (VEGF-A and WFDC2). (**B**) Comparison between the distributions of the abundance of the top five HGSC-associated snoRNAs in 18 SBT and 19 HGSC tissues. The abundance of the snoRNAs was determined as described in A and the data shown as a box plot. Each point in the box plot represents one tissue and the data obtained from HGSC and SBT are shown in purple and pink, respectively. The stars indicate *P*-values from Tukey tests from left to right of 0.002 (**), 0.002 (**), 0.02 (*), 0.02 (*) and 0.04 (*). (**C, D**) Identification of optimal snoRNA signatures of HGSC. Thirty-one different combinations of the five HGSC-associated snoRNAs were evaluated and the receiver operating characteristic (ROC) curve of the most sensitive (C) and specific (D) combinations are shown. The curves show the snoRNA combinations in grey. Combo XII is composed of SNORA19, SNORA56 and SNORA81, while Combo XXIII is composed of SNORA19, SNORA56, SNORA81 and SNORD72. The optimal discrimination cut-off is indicated by the red dot. The pie charts shown on the right indicate the signature dependent distribution of the 37 tissues examined. SBT and HGSC are indicated in pink and purple, respectively. Tissues that are correctly identified are indicated in black and dark grey, while tissues that are misidentified are indicated in light grey and white, respectively. (**E**) snoRNAs may discriminate between HGSC and SBT in a host gene independent manner. The capacity of each of the top five HGSC-associated snoRNAs to discriminate between HGSC and SBT tissues was compared to that of their host genes using ROC curves. The area under the curve (AUC) is indicated for each snoRNA (black) and each host gene (grey), while the dotted line indicates a random distribution. *P*-value for two-tailed tests were calculated for each ROC curve. (**F**) The expression of all but one HGSC-associated snoRNAs and their host genes is independently regulated. The abundance of the top 5 HGSC-associated snoRNAs and their host mRNAs in seven healthy tissues and two human and brain references was retrieved from published TGIRT-seq datasets (42,45) and presented as a scatter plot. The Pearson correlation for the different gene pairs is indicated below each graph.

To validate the predicted upregulation of the 14 differentially expressed snoRNAs in HGSC, we monitored their expression using RT-qPCR in the same tissues used for sequencing. As indicated in Supplementary Figure S3, the sequencing and RT-qPCR expression values are well correlated supporting the conclusion of the RNA-seq. To confirm the association of the snoRNAs with HGSC and evaluate their potential as possible biomarkers, we monitored their expression using RT-qPCR in an independent cohort of 19 HGSC and 18 SBT tissues. As indicated, in Figure 3A, five snoRNAs (SNORA81, SNORA56, SNORD72, SNORA6 and SNORA19) were mostly uniformly overexpressed in HGSC when compared with SBT and displayed similar or better association with high-grade ovarian cancer than the known mRNA markers VEGF-A and WFDC2. The expression of these five snoRNAs was significantly upregulated in all HGSC tissues tested as compared to SBT tissues with Benjamini-Hochberg adjusted *P*-values between 0.002 and 0.04 (Figure 3B).

To evaluate the potential role as biomarkers for these snoRNAs, we evaluated whether the combination of multiple snoRNAs would increase the sensitivity to discriminate between HSGC and SBT. To identify the optimal HGSC signature that best discriminates between HGSC and SBT with the highest sensitivity and accuracy, we evaluated the discrimination value of different snoRNA combinations. We examined 31 different combinations of snoRNAs (all combinations of 1, 2, 3, 4 and 5 snoRNAs amongst this group) resulting in the identification of the combination of SNORA56, SNORA19 and SNORA81 (Combo XII) as the best HGSC signature with a maximum sensitivity value of 0.8 and specificity of value of 0.75 (Figure 3C). The second-best combination included four snoRNAs (SNORA81, SNORD72, SNORA56 and SNORA19, Combo XXIII) with sensitivity and specificity values of 0.6 and 1.0, respectively. At the optimal cut-off, the three snoRNA signature accurately identified most HGSC tissues (16 true positives and 14 true negatives tissues) with only four false positives and three false negatives. In contrast, the four snoRNA signature resulted in zero false positives and seven false negatives, accurately identifying 12 true positives and 18 true negatives at the optimal cut-off. Furthermore, the three snoRNA signature could significantly discriminate (Fisher's exact test ***P-value 0.0002), through a clustered heatmap, between the set of 37 HGSC and SBT tissues (Supplementary Figure S4). We conclude that SNORA56, SNORA19 and SNORA81 form the optimal H/ACA snoRNA signature for the identification of HGSC tissues.

### Induction of snoRNA expression in HGSC is not linked to host gene expression

The newly identified HGSC-associated snoRNAs are all expressed as part of introns of protein coding genes. This raises the question of whether the association of these snoRNAs with HGSC signifies a direct link between snoRNAs and the tissue type or an indirect effect of HGSC dependent induction of their host gene. To discriminate between these two possibilities, we compared the expression of the five HGSC-associated snoRNAs with the expression of their host genes in HGSC and SBT tissues. As indicated in Figure 3E, in all cases except that of SNORA56, the snoRNAs discriminated between HGSC and SBT independently of their host gene expression. In the case of SNORA56, both snoRNA and host gene DKC1, which encodes the H/ACA snoRNA binding protein and pseudouridylase dyskerin, were able to discriminate between SBT and HGSC tissues. This is likely due to the shared function of H/ACA snoRNAs and this interacting core protein. This indicates that in most cases the abundance of snoRNAs in cells is not linked to the amount of the host gene mRNA. To evaluate the validity of this assumption we compared the expression levels of the HGSC-associated snoRNAs and their host genes in different unrelated tissues (Figure 3F) using previously published TGIRT-seq datasets (42,45). As indicated in Figure 3F, we found no correlation between the snoRNA and host gene mRNA abundance except in the case of SNORA56 / DKC1 gene pair. Once again, these data indicate that the expression of snoRNA and host gene is not strictly linked but depends mostly on the function of the host gene. We conclude that factors independent of host gene expression contribute to the increased abundance of a subset of H/ACA snoRNAs in HGSC.

### The snoRNA signature can identify high-grade ovarian cancer in an independent set of mixed tissues

**Figure 4.**
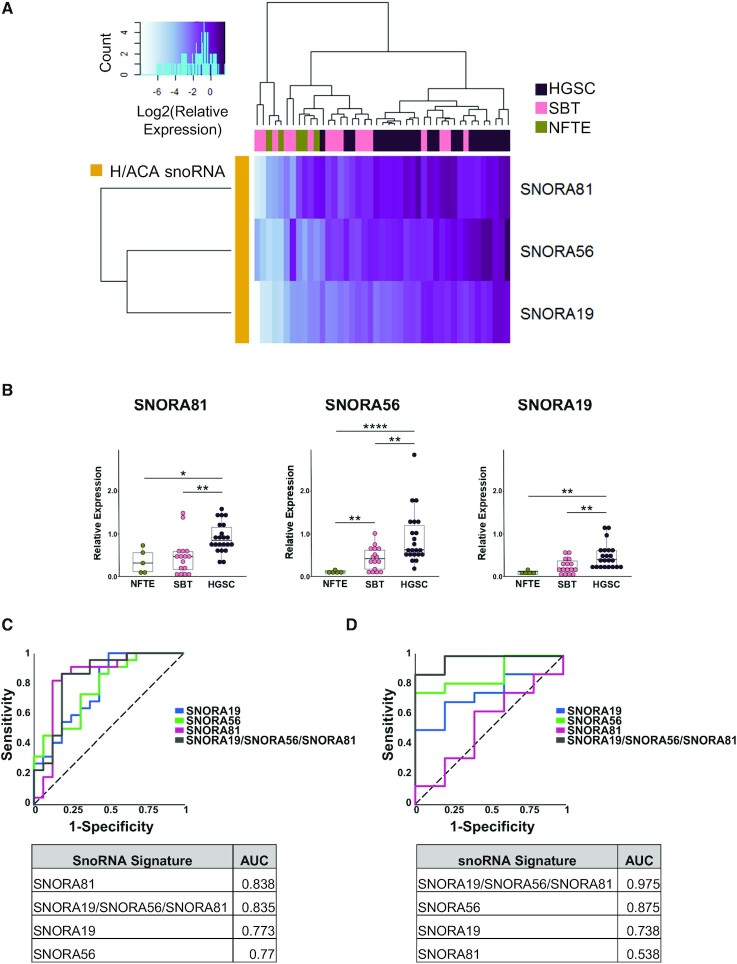
Validation of the snoRNA signature using independent set of normal, borderline, and high-grade tumour tissues. (**A**) The relative abundance of the three snoRNAs forming the HGSC signature identified in Figure 3 were examined in an independent mix of 43 tissues (22 HGSC, 16 SBT and 5 NFTE) using RT-qPCR. The log2 of the relative expression is presented as a clustered heatmap. The snoRNA names are indicated on the right and the identity of the tissue of origin is indicated on top. The key for the colored gradient is shown on the top left. (**B**) Comparison between the distributions of the abundance of the snoRNA from the signature in 5 NFTE, 16 SBT and 22 HGSC tissues. The abundance of the snoRNAs was determined as described in A. Each point in the box plot represents one tissue and the data obtained from NFTE, SBT and HGSC are shown in green, pink, and purple, respectively. The stars indicate *P*-values from Tukey tests from left to right of 0.04 (*), 0.002 (**), 0.004 (**), 8E-07 (****), 0.003 (**), 0.002 (**), 0.006 (**). (**C**) The snoRNA signature and its snoRNA components may discriminate between HGSC and SBT. On the top panel, the ROC curves of the discriminatory capacity of the three individual snoRNAs and the combination signature. On the bottom panel, the table of the area under the ROC curve (AUC) for individual snoRNAs and the combination. (**D**) The snoRNA signature improves the discrimination between SBT and NFTE tissues. The top and bottom panels are as described in C.

To evaluate the capacity of the snoRNA signature to identify HGSC, we challenged it using an independent set of 43 mixed SBT, HGSC and normal fallopian tube epithelial (NFTE) tissues. As indicated in Figure 4A, the signature was able to discriminate between HGSC, SBT and NFTE tissues. Interestingly, each snoRNA was also able to discriminate between these tissues individually albeit with varying accuracy (Figure 4B). For example, while SNORA81 was the best in discriminating between HGSC and SBT, SNORA56 was best in discriminating between NFTE and SBT (Figure 4C and D). Together the combination of the three snoRNAs presented the most stable and accurate solution for discriminating between NFTE, SBT and HGSC (Figure 4C and D). We conclude that the snoRNA signature is capable of not only distinguishing between SBT and HGSC but can also distinguish between NFTE and SBT.

### The increased expression of snoRNA is associated with increased rRNA modifications in high-grade ovarian cancer

**Figure 5.**
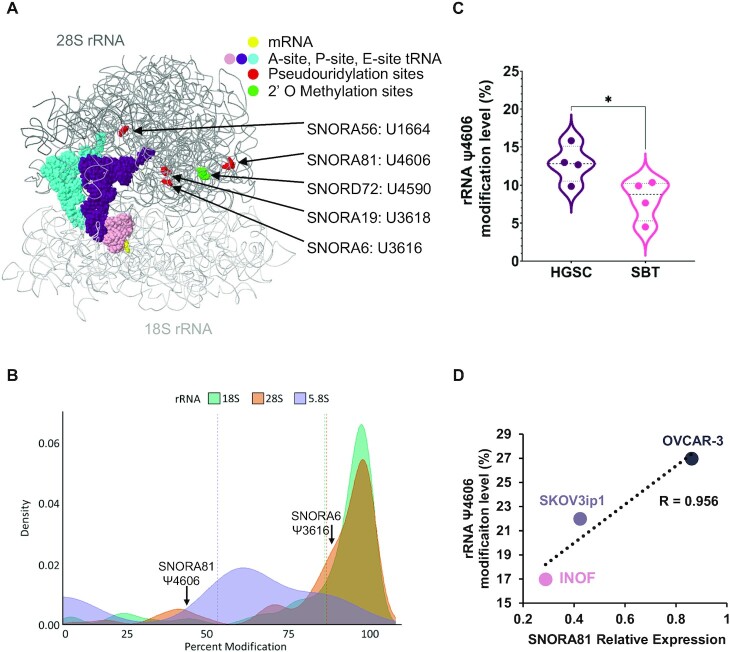
Ribosomal RNA pseudouridylation correlates with both SNORA81 expression and tumour aggressiveness. (**A**) Location of the rRNA pseudouridylation sites targeted by HGSC associated snoRNAs. The ribosome structure was generated as described in (32). SNORA19 targeted modifications at positions U3709 and U863 could not be illustrated in this view of the ribosome (**B**) Percent pseudouridylation of rRNA modification. The density plot indicates the previously reported percent modifications of all pseudouridylation sites in rRNA (50). The position of the only two partially modified sites in the 28S rRNA targeted by SNORA81 and SNORA6 are indicated on top. (**C**) SNORA81 targeted rRNA sequence displays increased levels of pseudouridylation in HGSC as compared to SBT. The violin plot compares the percent modification level of the SNORA81 targeted position 4606 of the 28S rRNA, in 4 HGSC and 4 SBT tissues. *T*-test was performed (*P-value 0.04) by comparing the rRNA modification level in HGSC and SBT. (**D**) The abundance of SNORA81 correlates with the level of its targeted rRNA modification and with its effect on cancer cell aggressiveness. The abundance of SNORA81 and the level of its targeted pseudouridylation were measured in the immortalized ovarian cancer cell line (INOF), the ascites driven serous cystadenocarcinoma cell line (SKOV3ip1) and high-grade serous ovarian cancer cell line (OVCAR-3). The Pearson correlation (*R*) value between the RNA abundance and level of pseudouridylation is indicated on the right.

Most H/ACA snoRNAs guide rRNA modifications and are tightly linked to changes in translation patterns (18,46,47). Consistently, 4 of the 6 nucleotides modified by the 5 HGSC-associated snoRNAs are located predominantly in the 28S rRNA near the peptide exit channel of the ribosome (Figure 5A) (42,45,48). Out of these four residues located in the 28S rRNA near the peptide exit channel, two (position 3616 targeted by SNORA6 and position 4606 targeted by the signature component SNORA81) are partially modified suggesting an important regulatory role (49,50) (Figure 5B). To determine whether changes in snoRNA expression correlate with changes in rRNA modification, we examined the modification level at position 4606, which is targeted by SNORA81, in 4 SBT and 4 HGSC tissues using a CMC-RT and ligation assisted PCR analysis of pseudouridylation (Ψ) modification (CLAP) assay (35). As indicated in Figure 5C, the site targeted by SNORA81 was more modified in HGSC than in SBT as would be expected from the increased expression of SNORA81 in these tissues. Consistently, we have also found a strong positive correlation between the expression of SNORA81 and the modification of its targeted 28S rRNA. As indicated in Figure 5D, both snoRNA and rRNA pseudouridylation levels were at the lowest level in a normal immortalized ovarian cell line (INOF) and highest in the high-grade model cell line (OVCAR-3). We conclude that the level of rRNA modification correlates with both tumour aggressiveness and snoRNA expression.

### Knockdowns of high-grade ovarian cancer associated snoRNAs inhibit cell growth

**Figure 6.**
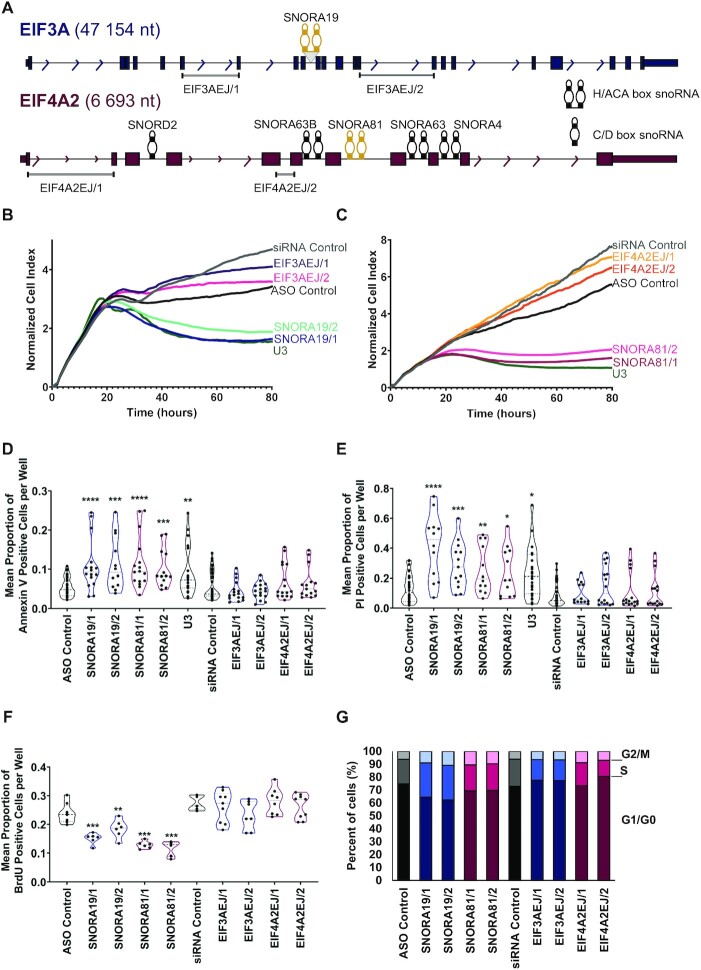
HGSC-associated snoRNAs induce cell survival and inhibit apoptosis. (**A**) SNORA19 and SNORA81 are expressed from host genes encoding the translation initiation factors EIF3A and EIF4A2 respectively. The structure of the two snoRNA encoding genes is depicted to scale. Exons, introns, and snoRNAs are indicated in blue (EIF3A) and purple (EIF4A2) boxes, lines and stem-loops, respectively. Both SNORA19 and SNORA81 are indicated in yellow. The names of the snoRNAs are indicated above them and the position of the exon-junction siRNAs used to inhibit the expression of the mature mRNA are shown at bottom. Arrows indicate the direction of transcription and gene sizes are shown on the left. (**B**) Knockdown of SNORA19 represses cell growth. The ovarian cancer model cell line SKOV3ip1 was transfected with two ASOs against SNORA19 (SNORA19/1 and SNORA19/2) or two siRNAs targeting the exon junction of EIF3A mRNA (EIF3AEJ/1 and EIF3AEJ/2) and the effect on growth was followed in real-time through changes in cell impedance. Unrelated siRNAs and ASOs were used as negative controls and an ASO against U3 snoRNA was used as a positive control. The y-axis indicates the normalized cell index, which is the average of the three technical replicates. (**C**) Knockdown of SNORA81 represses cell growth. Depletions of SNORA81 (SNORA81/1 and SNORA81/2) and its host gene (EIF4A2EJ/1 and EIF4A2EJ/2) were carried out as described in (B). (**D**) Knockdowns of SNORA19 and SNORA81 induce apoptosis. The effect of the different knockdowns described in B and C on apoptosis was evaluated 48 hours after transfection using Annexin V assays. The results, shown as violin plots, represent the mean proportion of annexin V positive cells per well. Mann-Whitney tests were performed and are indicated by asterisks. The stars indicate *P*-values from left to right for SNORA19/1 (****P-value < 0.0001), SNORA19/2 (***P-value 0.0009), SNORA81/1 (****P-value < 0.0001), SNORA81/2 (***P-value 0.0002) and U3 (**P-value 0.008), as compared to the control ASO. (**E**) Knockdowns of SNORA19 and SNORA81 induce necrosis. The experiment was carried out as in D, but necrosis caused by contact of Propidium iodide and nucleic acids in cells with compromised membranes was assessed. The stars indicate *P*-values from left to right for SNORA19/1 (****P-value < 0.0001), SNORA19/2 (***P-value 0.0004), SNORA81/1 (**P-value 0.01), SNORA81/2 (*P-value 0.04) and U3 (*P-value 0.02) as compared to the control ASO. (**F**) Knockdowns of both SNORA19 and SNORA81 inhibit cell proliferation. SKOV3ip1 were transfected as described in (B) and (C) and labelled with BrdU 48 h after transfection. The violin plots show the mean proportion of BrdU positive cells per well. Mann–Whitney tests were performed, and significance is indicated by asterisks. The stars indicate *P*-values from left to right for SNORA19/1 (***P-value 0.0007), SNORA19/2 (**P-value 0.008), SNORA81/1 (***P-value 0.0007), SNORA81/2 (***P-value 0.0007) as compared to the control ASO. (**G**) Knockdown of SNORA19 and SNORA81 causes arrest in the S phase of the cell cycle. SKOV3ip1 cells transfected and propidium iodide-stained were analyzed by FACS to determine their cell cycle phase after 48h and these proportions were displayed as stacked bar graphs. The cell cycle phases detected after the knockdown of SNORA19 and SNORA81 are shown in shades of blue and purple, respectively.

Two of the snoRNAs (SNORA19 and SNORA81) are embedded in the introns of genes encoding eukaryotic translation initiation factors EIF3A and EIF4A2 (Figure 6A). EIF3A harbours a single copy of SNORA19, while EIF4A2 introns contain SNORA81 along with four other non-HGSC-associated snoRNAs (SNORA4, SNORA63, SNORA63B and SNORD2). On the other hand, SNORA56 is embedded in an intron of the gene encoding the pseudouridine synthase DKC1 (Supplementary Figure S5A). The introns of DKC1 contain SNORA56 along with one other snoRNA (SNORA36A) which is not associated with HGSC. Accordingly, to evaluate the biological significance of the snoRNA signature, we knocked down SNORA19, SNORA56 and SNORA81 and examined the impact on cell viability. All three signature snoRNAs were first knocked down in SKOV3ip1 cell line, which features moderate snoRNA expression and rRNA modification. The knockdowns were performed using two independent antisense oligonucleotides (ASOs) which target the RNA for degradation using RNase H and the effect on cell viability examined (Supplementary Figures S5, S6 and Figure 6B and C). In the case of SNORA56 the knockdown of the snoRNA did not affect cell growth suggesting that while this snoRNA may function as a marker it does not directly contribute to cell growth under the condition tested (Supplementary Figure S5D). In contrast, the ASO targeting SNORA19 and SNORA81 significantly reduced cell growth to levels similar to that of the positive control U3 snoRNA, which is essential for rRNA processing (Figure 6B and C) (51). Since the ASO may target both the mature snoRNA and the snoRNA embedded in the intron of the host gene we also knocked down the host RNA of SNORA19 and SNORA81 using two independent siRNAs targeting the mature mRNA splice junction. All ASOs reduced both snoRNA and host mRNA levels as expected while the siRNAs reduced the host mRNA level without affecting the snoRNA abundance (Supplementary Figure S6A-C). SNORA19 knockdown altered both host mRNA and protein level, while SNORA81 knockdown altered only the host mRNA (Supplementary Figure S6D and 6E). These results indicate that while all three snoRNAs may function as HGSC markers only SNORA81 and SNORA19 may affect the viability of cancer cells.

### SNORA19 and SNORA81 are required for cells survival and proliferation

To understand the mechanism by which SNORA19 and SNORA81 affect cell growth we examined the impact of their knockdown on cell survival and proliferation rate, in different ovarian cancer model cell lines featuring different levels of snoRNA expression and rRNA modification. As indicated in Figure 6D and E, the knockdown of the snoRNAs and not of their host genes increased apoptosis and necrosis. This indicates that SNORA19 and SNORA81 promote cell survival by inhibiting apoptosis and cell death. Interestingly, the knockdown of the snoRNAs decreased cell proliferation and delayed the S phase of the cell cycle suggesting a role in promoting cell replication (Figure 6F and G). The effects of SNORA81 and SNORA19 on apoptosis, necrosis, proliferation, and cell cycle were observed in all three ovarian cancer model cell lines tested (Supplementary Figures S7 and S8). Consistently, the knockdown of SNORA19 and SNORA81 and not their host genes decreased the expression of the cell proliferation markers CCND1, IGF1R and SYK and induced the expression of the apoptotic markers HRK, TNF and TNFRSF9 (Supplementary Figure S9). The decrease in CCND1, IGF1R and SYK explains the snoRNA dependent accumulation of cells in the S phase (Figure 6G) since they affect both G1/S transition and G2 checkpoint (52–54). The snoRNA knockdown dependent upregulation of HRK, TNF and TNFRSF9 suggests that the snoRNA affect the extrinsic apoptotic pathway and Bcl-x associated pathway (55,56). We conclude that SNORA19 and SNORA81 are required for cell survival and proliferation.

### SNORA81 promotes cancer cell migration and invasion

**Figure 7.**
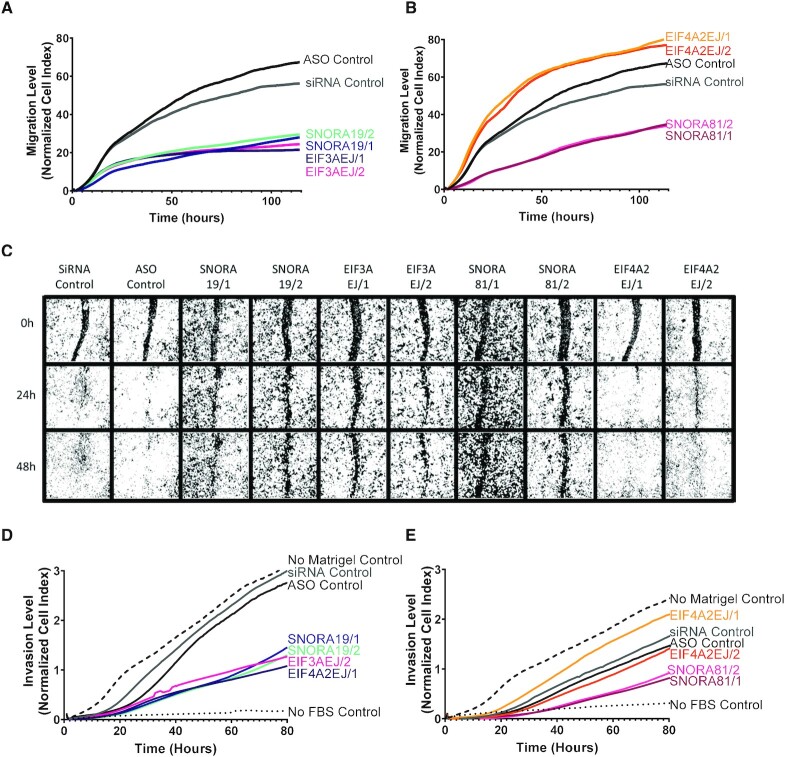
HGSC-associated snoRNAs promote cell migration. (**A, B**) Knockdown of SNORA19 (A) and SNORA81 (B) inhibits cell migration. snoRNA (SNORA19/1, SNORA19/2, SNORA81/1 and SNORA81/2) and their host genes (EIF3AEJ/1, EIF3AEJ/2, EIF4A2EJ/1 and EIF4A2EJ/2) were knocked down in SKOV3ip1 and cell migration monitored using Xcelligence RTCA after 24 h of transfection. A scrambled ASO (black) and a scrambled siRNA (grey) were used as controls. The y-axis indicates the normalized cell index, which is the average of the three technical replicates. The experiment was carried out in three biological replicates. (**C**) Knockdown of SNORA19 and SNORA81 delays wound healing. Cells were transfected as described in (A) and wounds introduced 48 h after transfection. Healing progress shown after 0, 24 and 48 h. (**D, E**) Knockdown of SNORA19 (D) and SNORA81 (E) inhibit cell invasion. Cells were transfected as described in A and cell invasion was monitored using Xcelligence RTCA CIM plate layered with matrigel 24 h after transfection. A scrambled ASO (black) and a scrambled siRNA (grey) were used as controls. No matrigel in upper chamber (dashed line) and no FBS in lower chamber (dotted lines) were used as controls. The y-axis indicates the normalized cell index, which is the average of the three technical replicates.

To better assess the contribution of HGSC-associated snoRNAs to cancer cell aggressiveness, we examined the impact of knocking down SNORA19 and SNORA81 on migration, wound healing, and invasion. The capacity of the cell to migrate was examined using CIM plates 24 h post-transfection with the ASOs and siRNAs targeting the snoRNAs or their host genes’ mature RNA, and the number of migrated cells measured in real time. As indicated in Figure 7A, the knockdown of SNORA19 and its host gene affected cell migration indicating at least in this case that the inhibition of cell migration is caused in part by the expression of EIF3A. In contrast, the knockdown of SNORA81 and not its host gene inhibited cell migration indicating that the effect on migration is specific to SNORA81 (Figure 7B). Similarly, the knockdown of SNORA81 and not SNORA19 inhibited the rate of wound healing in a host gene independent manner (Figure 7C). Indeed, while most cells either completely or partially healed the wound 48 hours post-scratching, those transfected with ASOs against SNORA81 did not (Figure 7C 81/1 and 81/2 bottom panel). The cell invasion was assessed similarly to the cell migration except a layer of Matrigel coating the upper chamber of the CIM plate permitted us to determine the cells’ invasion rate after the depletion of either the snoRNAs or the host genes. Similarly, to the cell migration assay, the knockdown of both SNORA19 and EIF3A inhibited cells’ invasion ability (Figure 7D), while only the knockdown of SNORA81 and not its host gene repressed cell invasion (Figure 7E). We conclude that HGSC-associated snoRNAs may promote cell migration and invasion in a host independent manner at least in the case of SNORA81.

### SNORA81 knockdown inhibits the modification and the accumulation of the 28S rRNA

Since knocking down SNORA81 and SNORA19 had similar effects as knocking down the principal rRNA processing snoRNA U3, we hypothesized these snoRNAs could affect cell invasiveness through changes in rRNA biogenesis. Accordingly, we examined the impact of knocking down SNORA81 on the modification and biogenesis of the 28S rRNA. Unlike SNORA19, SNORA81 has only one rRNA target that is more modified in HGSC than SBT (Figure 5C). As shown in Supplementary Figure S10A and B, the knockdown of SNORA81 reduced the modification in position 4606 of the 28S rRNA and reduced the amount of the 28S rRNA leading to changes in the ratio of 28S/18S rRNA, as would be expected from defects in the biogenesis of the large ribosomal subunit. The level of RNA modification was calculated relative to the total amount of RNA and as such is not affected by changes in rRNA amount. This result links snoRNA expression to rRNA modification and abundance. We conclude that SNORA81 promotes cell aggressiveness at least in part through the modulation of rRNA modification and biogenesis.

## DISCUSSION

In this study, we identified five different snoRNAs that distinguish between HGSC and SBT with high specificity and accuracy (Figure 3). Interestingly, the knockdown of two examples of these HGSC-associated snoRNAs, SNORA81 and SNORA19, inhibited the proliferation and induced apoptosis of three different model ovarian cancer cell lines consistent with their upregulation in the aggressive HGSC (Figure 6 and Supplementary Figure S8). More importantly, the reduction of these two HGSC-associated snoRNAs inhibited cell migration, wound healing and cell invasion underlining their contribution to the biology of HGSC and tumour aggressiveness. Together the data obtained here suggest a new role for snoRNAs in the promotion of tumour aggressiveness.

Development of cancer, and in particular the increased proliferation rate of cancer cells, is often linked with the dysregulation of translation and ribosome biogenesis (57). Indeed, the increased production of ribosomes is a pre-requisite for increased cell proliferation (58). Accordingly, we hypothesized that the highly invasive and fast growing HGSC cells would also require an overall upregulation of the ribosome production machinery when compared with the much slower growing SBT counterpart. Surprisingly, however, our data indicate that the expression of snoRNAs, key effectors in ribosome biogenesis, is not universally upregulated in HGSC (Figure 2). In fact, the majority of C/D snoRNAs had the tendency to be slightly under expressed in HGSC when compared with SBT. This clearly indicates that the degree of cancer aggressiveness is not strictly linked to the overall induction of snoRNAs. Furthermore, the clear difference in the number of the methylation (C/D) and pseudouridylation (H/ACA) guide snoRNAs that are specifically upregulated in HGSC suggests that these two classes of snoRNAs play distinct roles in the development or maintenance of HGSC. Indeed, our work suggests that H/ACA snoRNAs have higher propensity for upregulation in HGSC and are as such more likely to function as positive regulators of cancer cell proliferation and survival. This is consistent with earlier results linking the upregulation of SNORA7B with breast cancer (59) and SNORA42 in lung cancer (60). The link between the upregulation of H/ACA snoRNAs and poor prognosis was also observed in the case of non-small cell lung cancer, where expression of the H/ACA snoRNA binding protein Nop10 was found to drive tumorigenesis (61). However, even within the H/ACA class of snoRNAs only 14% were upregulated indicating once more that H/ACA snoRNA expression is not randomly dysregulated in HGSC (Figure 2). Instead, it appears that HGSC requires increased production of a specialized subgroup of H/ACA snoRNAs. The gene specific contribution of H/ACA snoRNAs to tumour aggressiveness is also evident from a previous study showing that SNORA24 may function as a suppressor of hepatocellular carcinoma and its reduced expression is associated with poor prognosis of hepatocellular carcinoma (62). As such, it appears that H/ACA snoRNAs may influence carcinogenesis in different ways depending on the identity of the snoRNA and cancer type.

The potential of pseudouridylation dependent modification was also shown in different model systems and in cancer cells (63). For example, knockdown of SNORA24 was shown to alter ribosome dynamics leading to increased miscoding and stop codon read through. Furthermore, knockdowns of several H/ACA snoRNAs were shown to alter translation accuracy in yeast (46,64). In this study we provide a possible mechanism of H/ACA snoRNA function in increasing tumour aggressiveness through promoting the modification and biogenesis of rRNA. Indeed, the results shown in Figure 6 indicate a link between rRNA modification and tumour aggressiveness. Changing the level of rRNA modification may reduce RNA stability through destabilizing its interaction with ribosomal proteins and biogenesis factors. The exact mechanism by which H/ACA snoRNAs may modify ribosome function will become clearer as more examples of cancer associated snoRNAs are identified. Meanwhile, the data presented here provide a possible mechanism where increased expression of a subclass of H/ACA snoRNAs may promote tumour aggressiveness through the modulation of rRNA modification and biogenesis.

## DATA AVAILABILITY

RNA-seq data used in this study are available through the NCBI Gene Expression Omnibus (GEO; https://www.ncbi.nlm.nih.gov/geo) under the accession number GSE181496.

## SUPPLEMENTARY DATA

Supplementary Data are available at NAR Cancer Online.

## Supplementary Material

zcab050_Supplemental_Files
