# Supplementary material for: High-grade ovarian cancer associated H/ACA snoRNAs promote cancer cell proliferation and survival
Source: NAR Cancer. 2022 Jan 14;4(1):zcab050. doi: 10.1093/narcan/zcab050 (PMC8759569; doi:10.1093/narcan/zcab050)
Supplement: zcab050_Supplemental_Files [file zcab050_supplemental_files.zip › Faucher-Giguere et al., sup figures_V7.pdf]

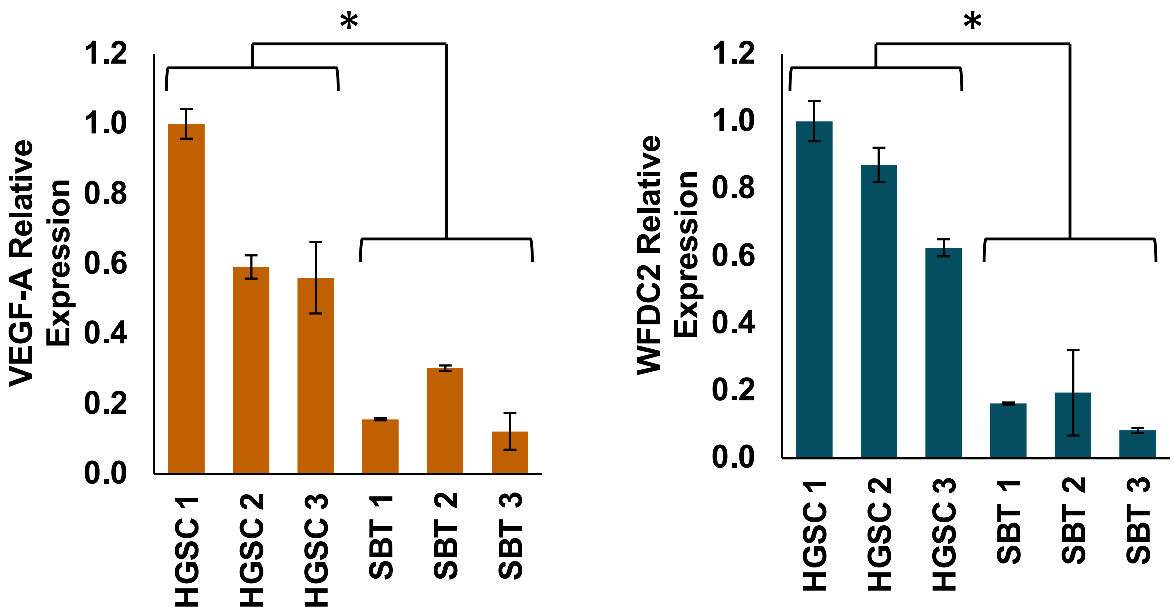


**Supplementary Figure 1 (related to Figure 1). Expression levels of the high-grade molecular markers VEGF-A and WFDC2 in the HGSC and SBT tissues used for sequencing in Figure 1.** The abundance of VEGF-A and WFDC2 was determined using RT-qPCR in the 6 tissues that were used for NF-TGIRT-seq. The stars indicate p-values of 0.04 (left panel) and 0.02 (right panel) as determined using T-tests.

**A**

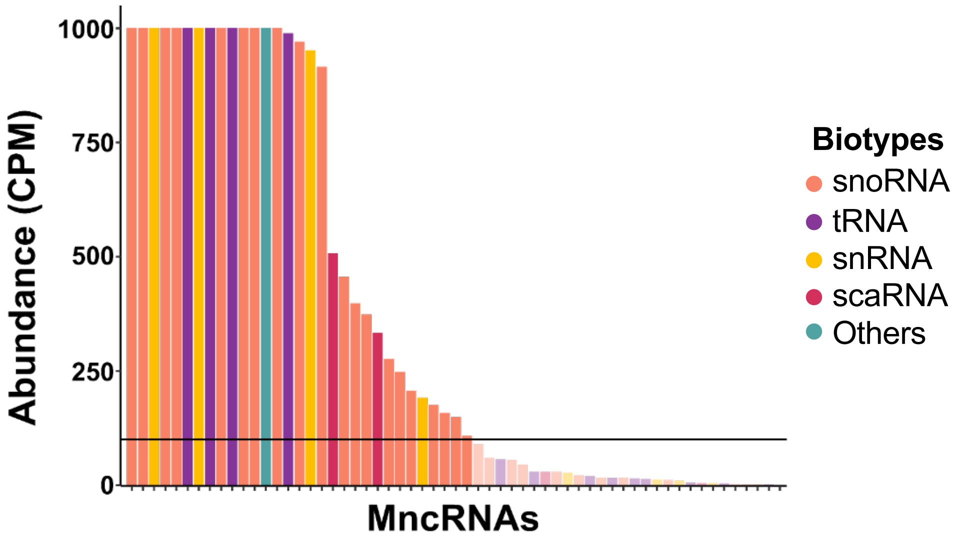


**B**

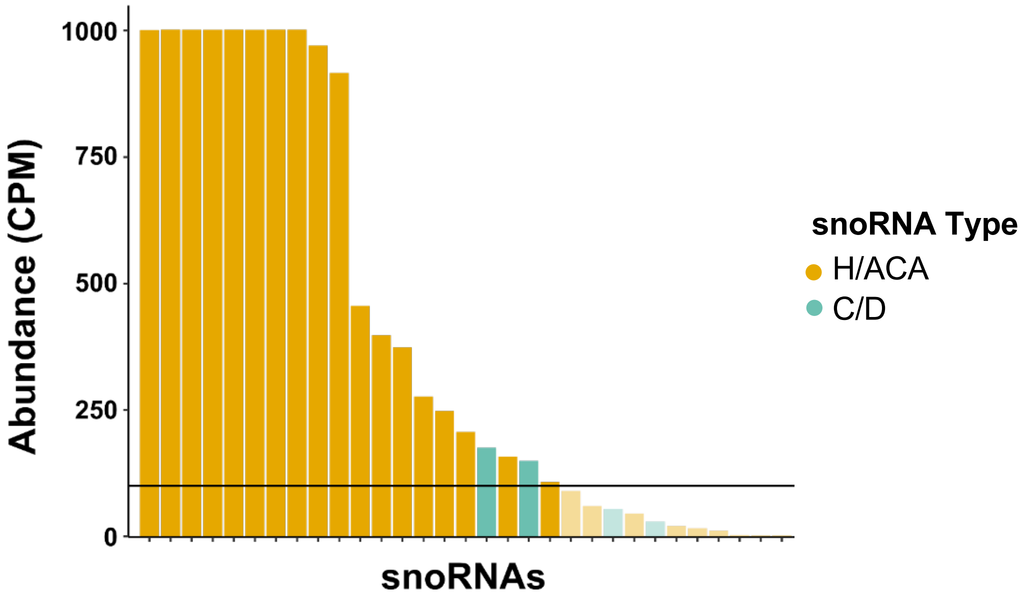


**Supplementary Figure 2 (related to Figures 1 and 2). Distribution of mncRNA abundance in SBT and HGSC tissues.** The highest number of counts per-million (CPM) detected for mncRNA (**A**) or snoRNA (**B**) in the 3 SBT and 3 HGSC tissues sequenced is plotted and the cut-off (100 CPM) used to select the RNA considered for the selection of potential HGSC markers is indicated by the horizontal line.

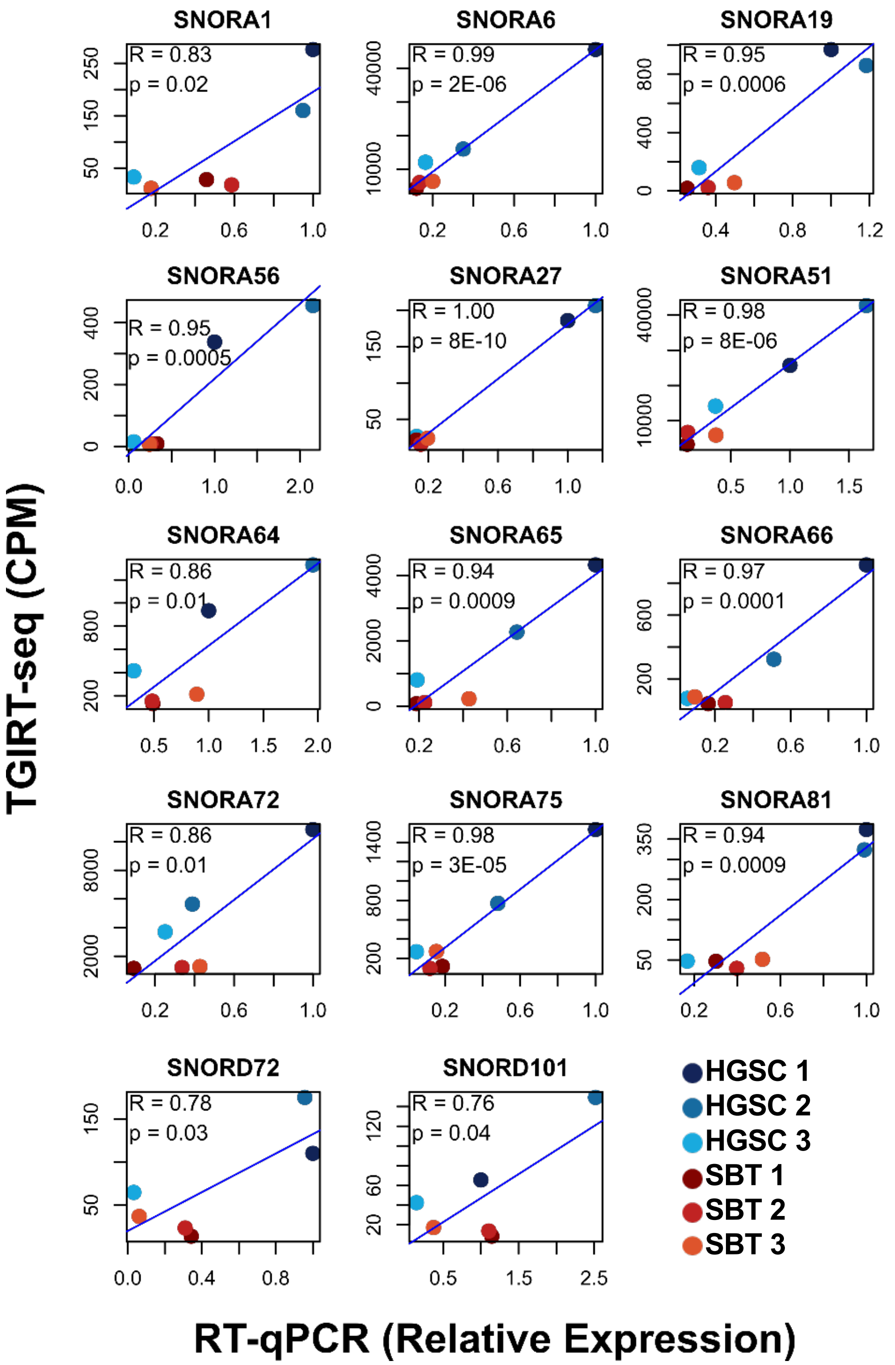


**Supplementary Figure 3 (related to Figures 1 and 2). Validation of the NF-TGIRT-seq identified HGSC-associated snoRNAs.** The abundance of the 14 snoRNAs that were identified by sequencing was examined using RT-qPCR and the results compared to those obtained by sequencing. Each point represents one tissue and those obtained from HGSC and SBT tissues are shown in blue and red shades, respectively. The Pearson correlation is indicated in the top left corner of each graph.

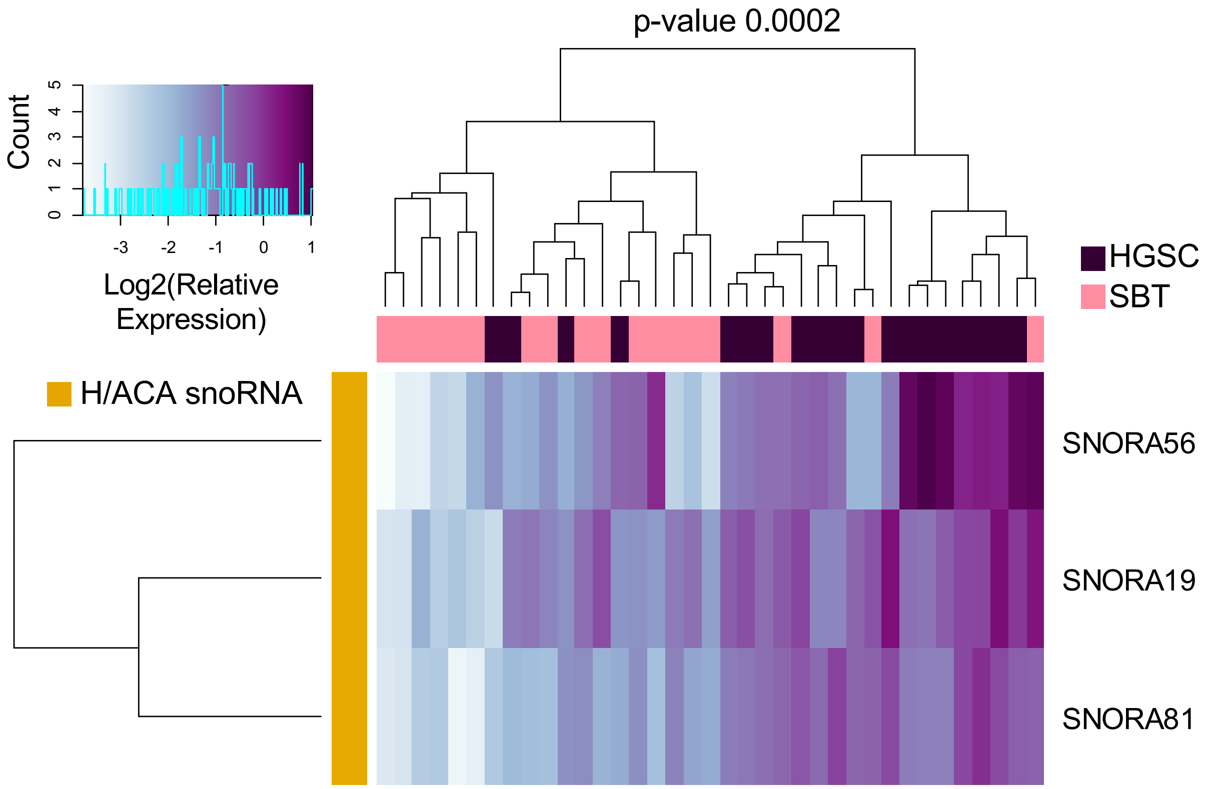


**Supplementary Figure 4 (related to Figure 3). Distribution of the HGSC snoRNA signature in different HGSC and SBT tissues.** The relative abundance of the three snoRNAs forming the best HGSC signature identified in Figure 3 were examined in 37 tissues using RT-qPCR. Data were normalized relative to the mean amount of MRPL19 mRNA and a spike-in of E. coli 16S and 23S rRNA and the log2 of the relative expression is presented as a clustered heatmap. The snoRNA names are indicated on the right and the identity of the tissue of origin is shown above. The color key histogram is shown on the top left, where the number of tissues (count) is shown for the color gradient. The Fisher's exact test was calculated for the capacity of the snoRNAs to discriminate between HGSC and SBT and shown on top.

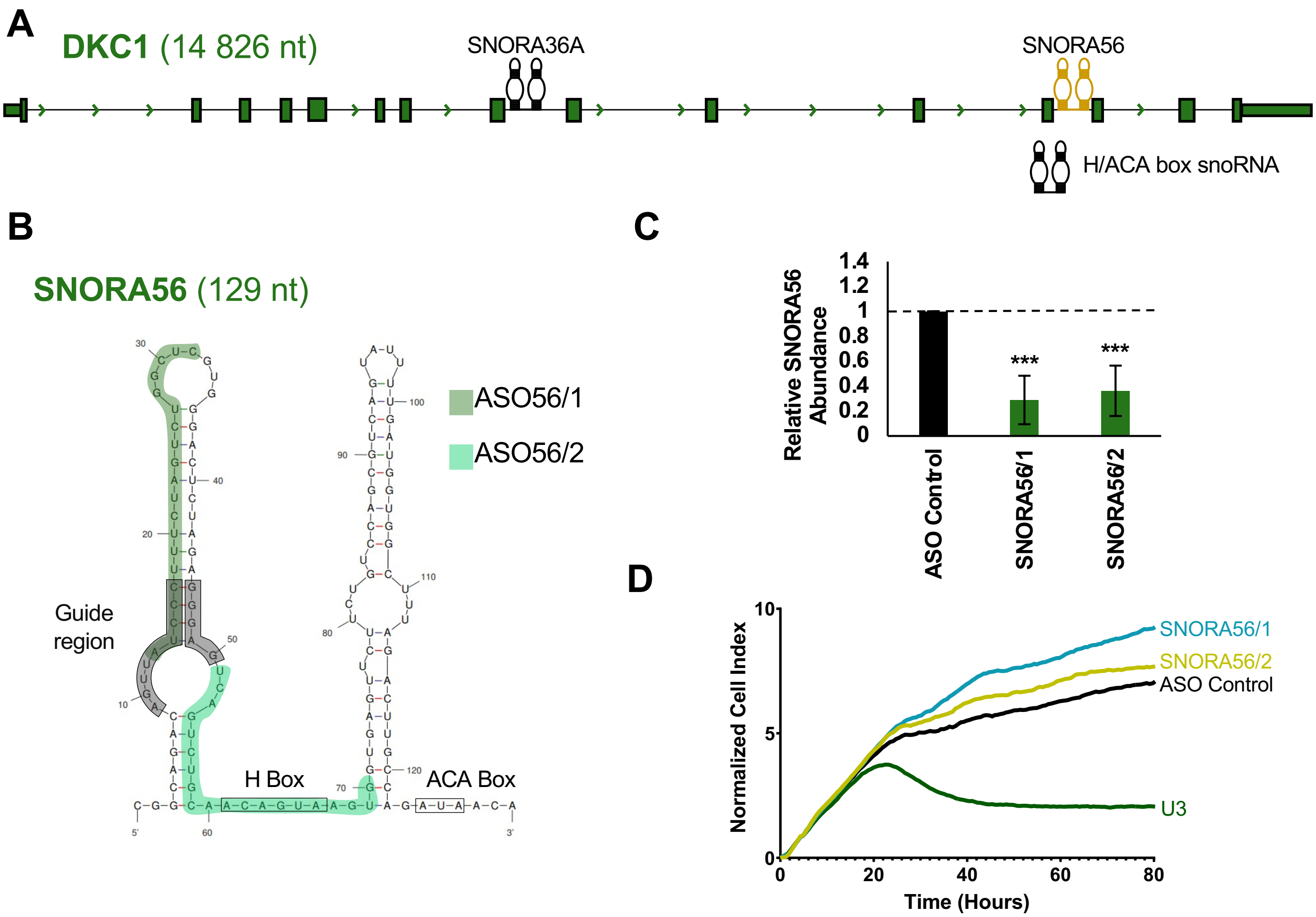


**Supplementary Figure 5 (related to Figure 6). Design, validation and effect of SNORA56 knockdown. (A)** SNORA56 is expressed from the intron of the gene coding for the pseudouridine Synthase DKC1. The structure of the snoRNA encoding gene is depicted to scale. Exons, introns, and snoRNAs are indicated in green boxes, lines and stem-loops, respectively. SNORA56 is indicated in yellow. The names of the snoRNAs are indicated on top. Arrows indicate the direction of transcription and gene size indicted on the top left. **(B)** Position of the ASOs used for the knockdown within the predicted secondary structure of SNORA56. The positions of the H and ACA boxes, the guide regions and ASOs are highlighted by the grey and green boxes, respectively. **(C)** The relative abundance of SNORA56 was determined using RT-qPCR after knockdown using two independent ASOs against the snoRNA. The standard deviation obtained from three technical replicates is shown in the form of error bars. Stars indicate p-values determined using T-tests where *** indicates p-value <0.001. **(D)** Knockdown of SNORA56 does not affect cell growth. The ovarian cancer model cell line SKOV3ip1 was transfected with two ASOs against SNORA56 (SNORA56/1 and SNORA56/2) and the effect on growth was followed in real-time through changes in cell impedance. Unrelated ASO was used as negative control and an ASO against U3 snoRNA was used as a positive control. The y-axis indicates the normalized cell index, which is the average of the three technical replicates.

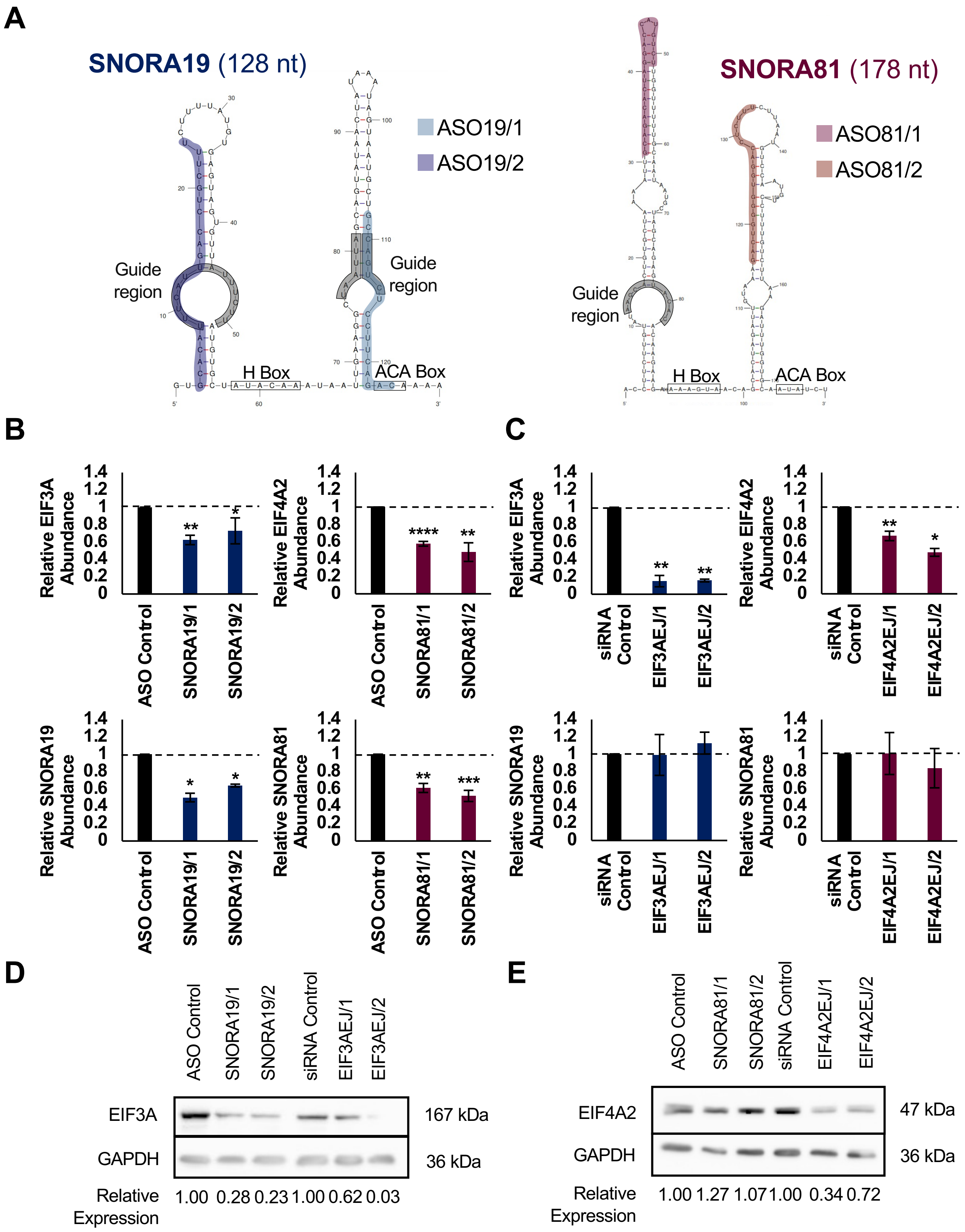


**Supplementary Figure 6 (related to Figures 6-7). Design and validation of SNORA19 and SNORA81 knockdowns. (A)** The structures of SNORA19 and SNORA81 and the positions of the H and ACA boxes, the guide regions and ASOs are highlighted by the grey and blue/purple boxes, respectively.

**Supplementary Figure 6 (continued) (B and C)** The relative abundance of EIF3A and EIF4A42 mRNA (upper panels) and SNORA19 and SNORA81 (lower panels) were determined using RT-qPCR after knockdown using two independent ASOs against each snoRNA (B) and two independent siRNAs against the mature splice junction sequence of each host mRNA (C). The standard deviation obtained from three technical replicates is shown in the form of error bars. *, **, *** and **** indicate p-values (determined by T-test) of 0.04, 0.002, 0.0008, 0.0001, respectively. **(D and E)** Effect of snoRNA and host gene knockdown on the abundance of host protein. The level of EIF3A and EIF4A2 proteins was determined using antibodies specific to each protein before and after knockdown of the snoRNAs and host genes. GAPDH was used as loading control and relative protein levels were quantified and indicated at the bottom. The siRNA and ASO used are indicated on the top and the position of each protein and estimated molecular weight are indicated on each side of the gel.

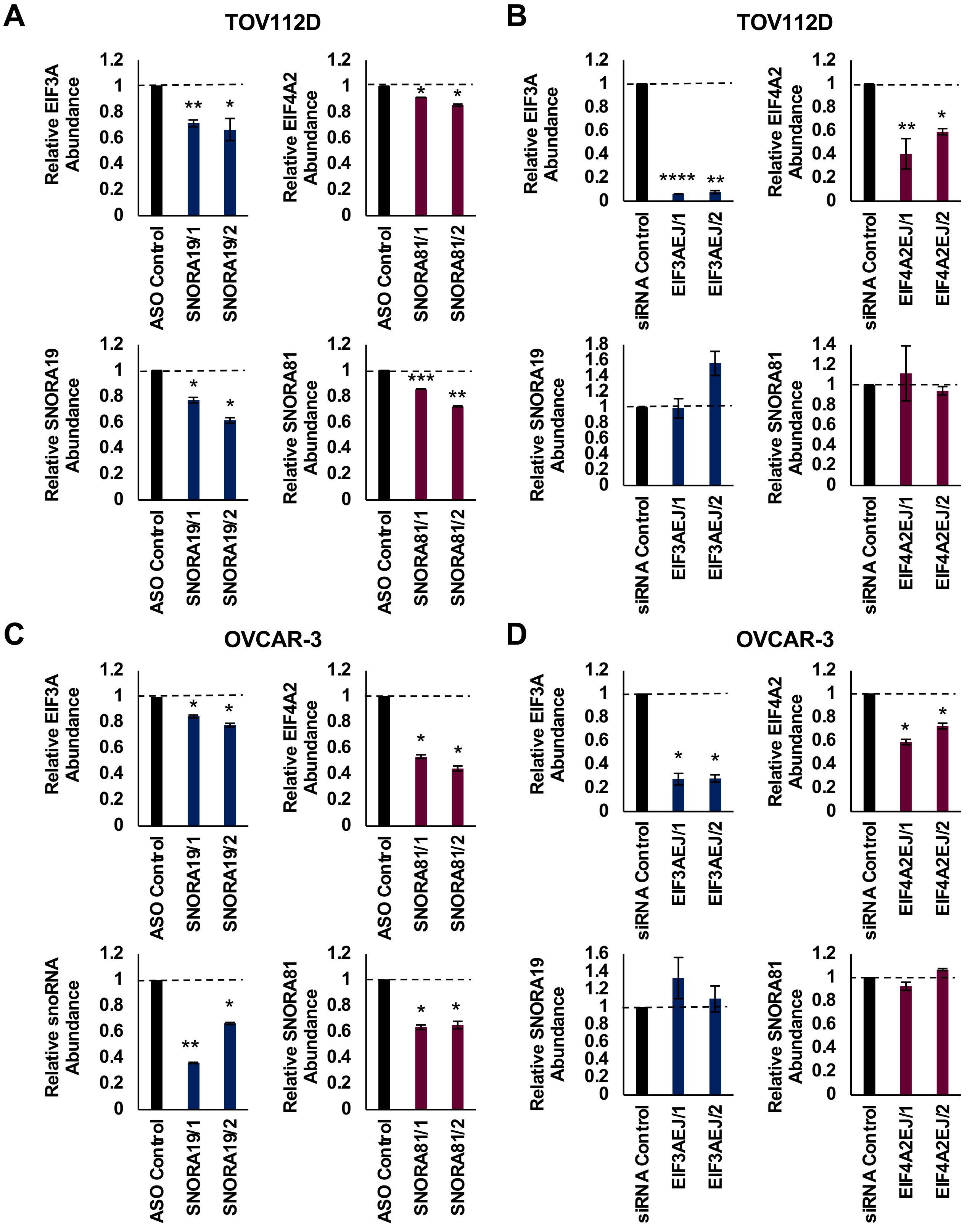


**Supplementary Figure 7 (related to Figure 6). Validation of SNORA19 and SNORA81 knockdowns in different ovarian model cell lines. (A and B)** Effect of the snoRNA (A) and their host gene (B) knockdowns on RNA abundance in TOV112D cell line.

**Supplementary Figure 7 (continued)** The abundance of the host mRNA (upper panels) and snoRNA (lower panels) was determined using RT-qPCR before and after knockdown of the snoRNAs (left panels) or their host genes (right panels) as described in supplementary Figure 6. Stars indicate p-values determined using T-tests where * are p-value <0.05, ** are p-value <0.01, *** are p-value < 0.001 and **** are p-value <0.0001. **(C and D)** Effect of the snoRNA (C) and host gene (D) knockdowns on RNA abundance in OVCAR-3 cell line. The effect of knockdown on the snoRNA and host mRNA abundance in the ovarian model cell line OVCAR-3 was determined and illustrated as described in A. Stars indicate p-values determined using T-tests where * are p-value <0.05, ** are p-value <0.01.

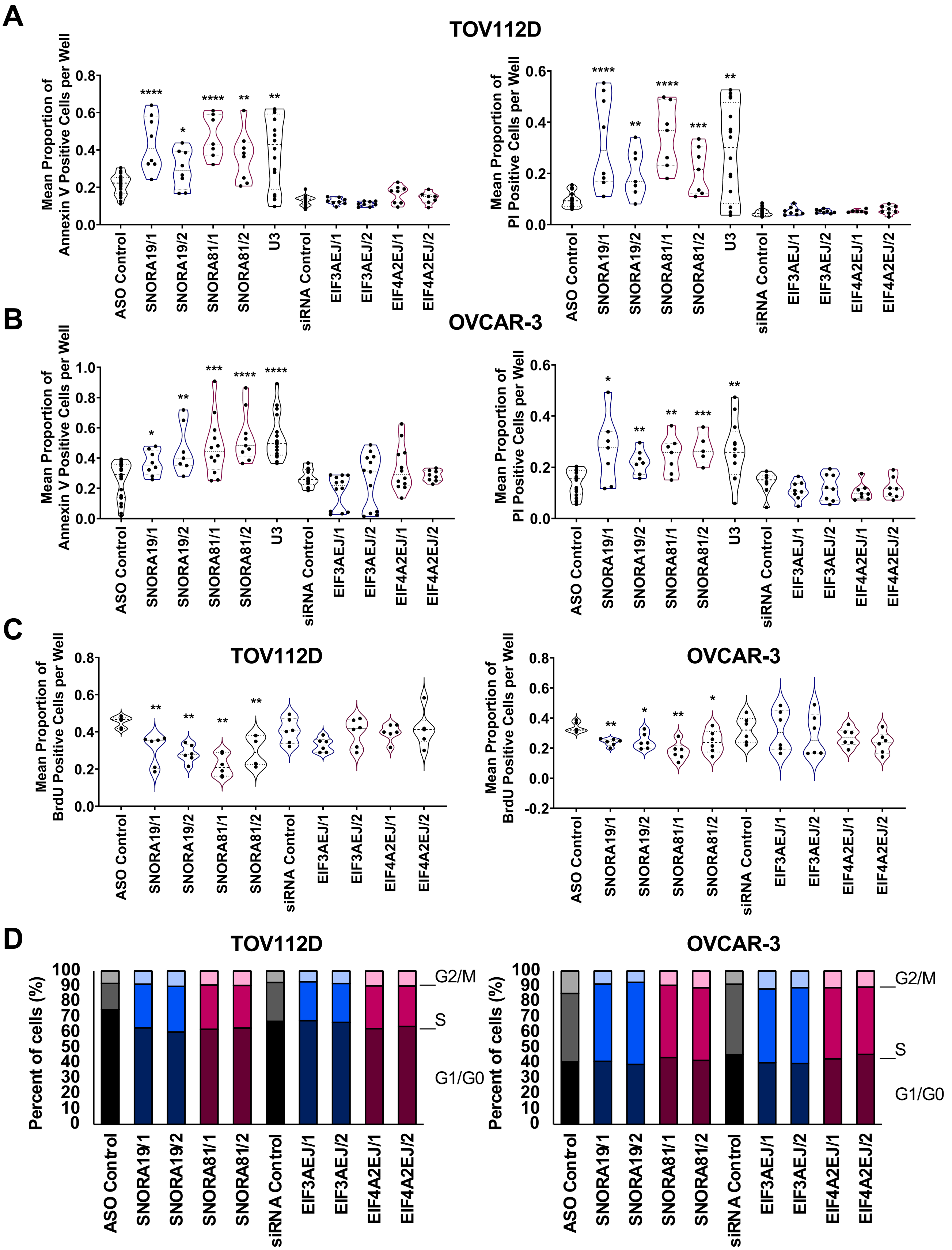


**Supplementary Figure 8 (related to Figure 6). HGSC-associated snoRNAs inhibit apoptosis and necrosis in different ovarian cancer cell lines.**

**Supplementary Figure 8 (continued) (A)** Knockdowns of SNORA19 and SNORA81 induce apoptosis (left panel) and necrosis (right panel) in TOV112D cell line. The snoRNAs and their host genes were knocked down and their effect on apoptosis (annexin V level) and necrosis (propidium iodide level) determined as described in Figure 6. Stars indicate p-values determined using Mann Whitney test where * are p-value <0.05, ** are p-value <0.01, *** are p-value < 0.001 and **** are p-value <0.0001. **(B)** Knockdowns of SNORA19 and SNORA81 induce apoptosis (left panel) and necrosis (right panel) in OVCAR-3 cell line. The effect of snoRNA and host gene knockdowns on apoptosis and necrosis in OVCAR-3 was determined and illustrated as described in A. Stars indicate p-values determined using Mann Whitney test where * are p-value <0.05, ** are p-value <0.01, *** are p-value < 0.001 and **** are p-value <0.0001. **(C)** Knockdowns of SNORA19 and SNORA81 inhibit cell proliferation in the TOV112D (right panel) and in the OVCAR-3 (left panel) cell lines. The snoRNA and host gene knockdowns and proliferation assay was performed as described in Figure 6F. Stars indicate p-values determined using Mann Whitney test where * are p-value <0.05, and ** are p-value <0.01. **(D)** Knockdowns of SNORA19 and SNORA81 cause arrest in S phase of the cell cycle in the TOV112D (right panel) and in the OVCAR-3 (left panel) cell lines. The knockdown and cell cycle assay were performed as described in Figure 6G.

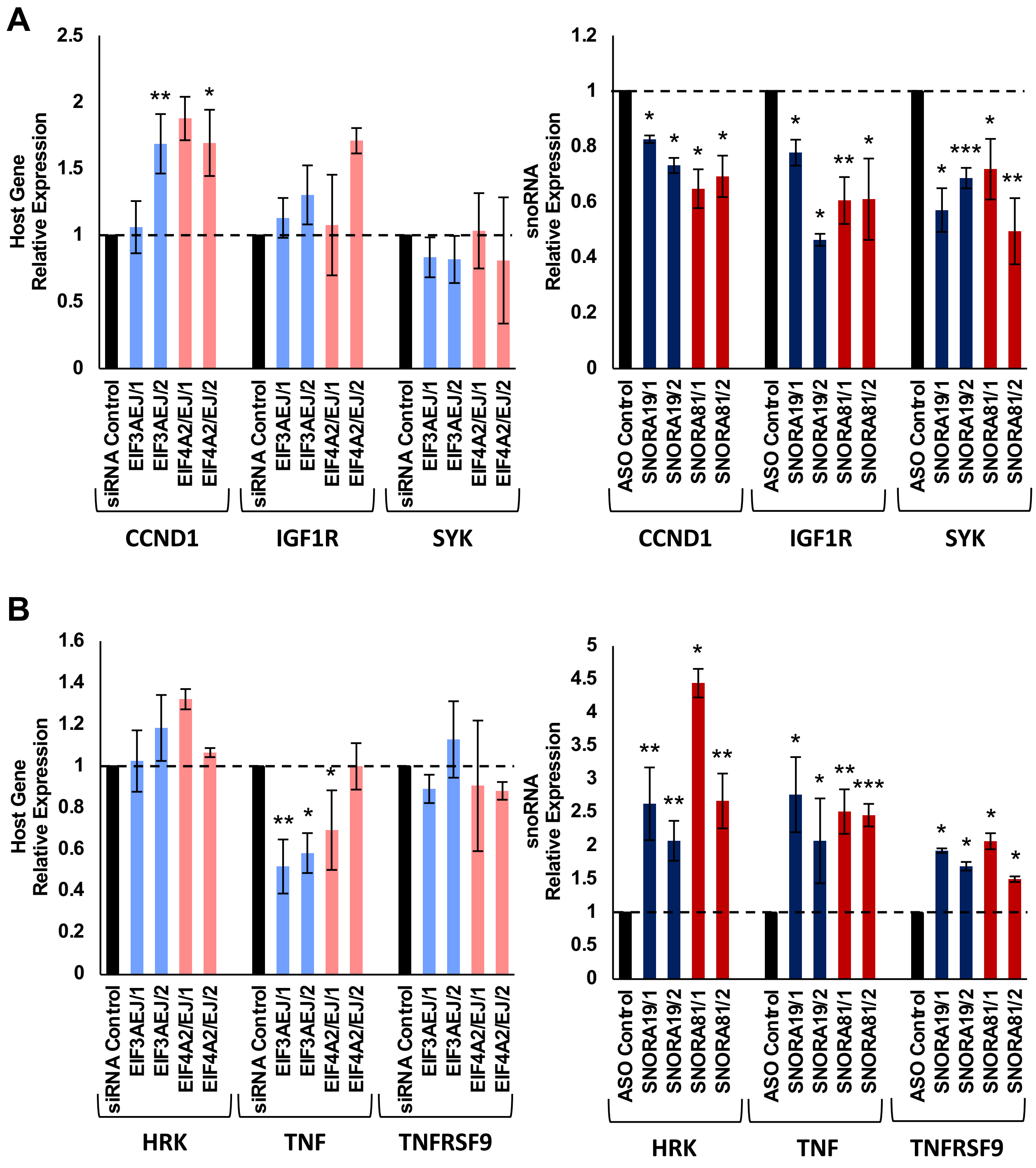


**Supplementary Figure 9 (related to Figure 6). SNORA19 and SNORA81 alter the expression of known cell cycle and apoptotic markers. (A)** Knockdown of SNORA19 and SNORA81 inhibits the expression of known cell cycle marker. The bar graph shows the relative abundance of the cell proliferation markers CCND1, IGF1R and SYK as determined by RT-qPCR before and after SNORA19 and SNORA81 knockdowns. In the left panel, the black, light blue and light red indicate RNA levels in cells transfected with the control siRNA, the EIF3A siRNAs and EIF4A2 siRNAs, respectively.

**Supplementary Figure 9 (continued)** In the right panel the black, dark blue and dark red indicate RNA levels in cells transfected with the control ASO, the SNORA19 ASOs and SNORA81 ASOs, respectively. Stars indicate p-values determined using T-tests where *, **, *** indicate p-value <0.05, <0.01 and < 0.001, respectively. **(B)** Knockdown of SNORA19 and SNORA81 induce the expression of apoptotic markers. The bar graph shows the relative abundance of the apoptotic markers HRK, TNF and TNFRSF9 as determined using RT-qPCR before and after the snoRNA knockdown. In the left panel the black, light blue and light red indicate RNA levels in cells transfected with the control siRNA, the EIF3A siRNAs and EIF4A2 siRNAs, respectively. In the right panel the black, dark blue and dark red indicate RNA levels in cells transfected with the control ASO, the SNORA19 ASOs and SNORA81 ASOs, respectively. Stars indicate p-values determined using T-tests where *, **, *** indicate p-value <0.05, <0.01 and < 0.001, respectively.

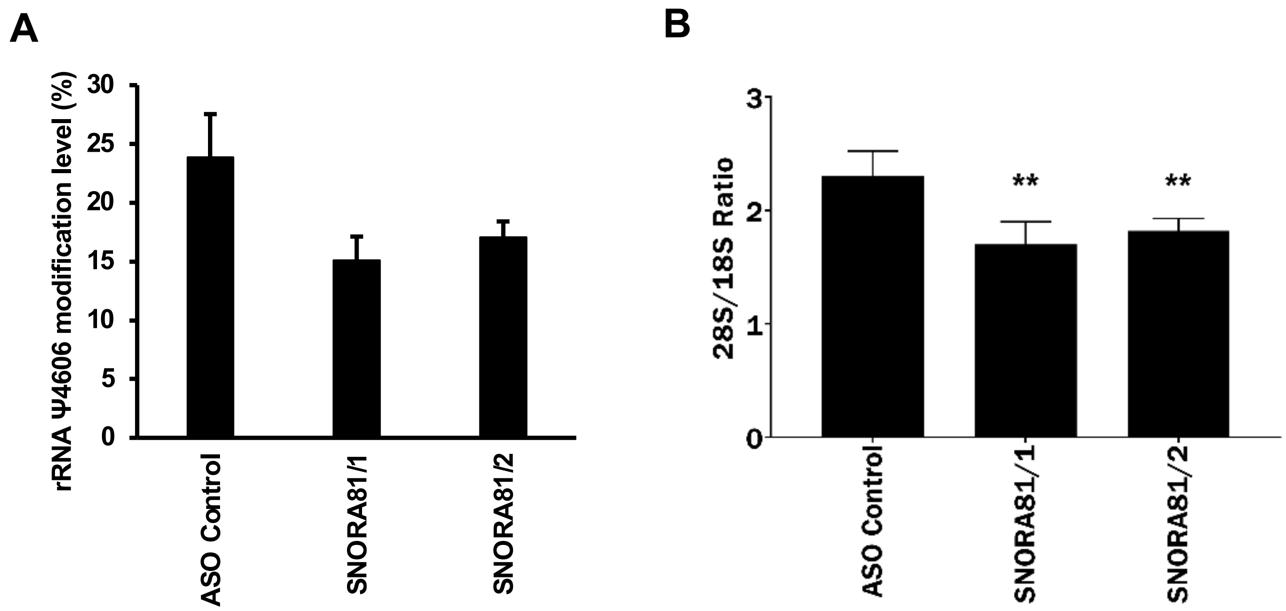


**Supplementary Figure 10 (related to Figure 5 and 6). Knockdown of SNORA81 inhibits its targeted rRNA pseudouridylation and the accumulation of the 28S rRNA. (A)** The level of Ψ4606 rRNA modification is decreased 48h after SNORA81 knockdown. CLAP assays were performed 48h after the snoRNA knockdown and the percent modification was calculated as described in the methods section. **(B)** Ribosomal RNA was detected using capillary electrophoresis in both ASO control and SNORA81 knockdown and the ratio of the 28S/18S rRNA shown in the form of a bar graph. T-tests were performed where ** indicate a p-value of ≤ 0.01.
